# Transdermal Delivery of Chinese Medicinal Formula Mitigates Pediatric Constipation by Modulating Intestinal Endocrine and Metabolic Homeostasis

**DOI:** 10.34133/bmr.0374

**Published:** 2026-06-10

**Authors:** Fengyuan Song, Yunhao Ren, Ming Zhu, Yuling Liu, Siping Wei, Hui Li, Lihua Peng

**Affiliations:** ^1^ School of Pharmacy, Southwest Medical University, Luzhou 646009, Sichuan, PR China.; ^2^ College of Pharmaceutical Sciences, Zhejiang University, Hangzhou 310058, Zhejiang, PR China.; ^3^ Jinhua Institute of Zhejiang University, Jinhua 321299, Zhejiang, PR China.; ^4^ Institute of Chinese Materia Medica, China Academy of Chinese Medical Sciences, Beijing 100700, PR China.; ^5^ Institute of Traditional Chinese Medicine Health Industry, China Academy of Chinese Medical Sciences, Nanchang 330115, Jiangxi, PR China.

## Abstract

Pediatric constipation, attributed to the functional immaturity of the gastrointestinal tract in children, is a common clinical disorder characterized by impaired gastrointestinal motility and infrequent bowel movements. Existing therapeutic strategies are frequently constrained by suboptimal efficacy owing to their single-target mechanisms and systemic toxicity. In contrast, Chinese medicinal formulas, with their multicomponent and multitarget intervention strategies, offer a highly suitable alternative for managing constipation. The traditional Chinese medicine formula “YiNianJin” (YNJ) is composed of cinnabar, rhubarb, stir-fried morning glory seeds, areca nut, and ginseng. It has demonstrated significant therapeutic efficacy in accelerating intestinal peristalsis through the synergistic regulation of aquaporin expression and the release of endocrine homeostatic transmitters. However, the clinical application of YNJ is significantly limited by conventional oral administration, which leads to Hg^2+^ accumulation and the low bioavailability of the active components. To address these challenges, we developed a sustained-release transdermal patch named YNJ patch (YNJP), which encapsulates cinnabar-loaded nanovesicles along with other active constituents in carboxymethyl cellulose sodium matrix. YNJP was shown to significantly enhance intestinal motility (as evidenced by a 24.02% increase in propulsion rate) by regulating the expression of mucin 2, aquaporin 3, and tight junction protein 1, while simultaneously promoting the release of endocrine homeostatic transmitters and *Lactobacilli*-mediated short-chain fatty acids. Therefore, YNJP is shown as a novel transdermal platform that alleviates pediatric constipation by modulating intestinal endocrine and metabolic homeostasis, enabling safe delivery of complex formulas with strong clinical potential for complex disorders.

## Introduction

Pediatric constipation, attributable to the structural immaturity of the gastrointestinal (GI) tract in children, represents a prevalent clinical condition characterized by dysregulated GI motility-mediated poor bowel movements, dry, and hard stools in children [1]. This condition is often accompanied by intestinal wall damage [2], reflecting its multifunctional disorder. However, current therapeutic strategies—particularly those relying on single-target mechanisms or involving systemic drug accumulation—often suffer from limited efficacy and pose potential hazards to children with underdeveloped organs. Chinese medicinal formulas exhibit distinct advantages due to their multicomponent and multitarget therapeutic mechanisms [3]. Such drugs not only enhance therapeutic efficacy through complementary pharmacological actions but also minimize adverse effects in pediatric populations by reducing the required dosage of individual constituents. Moreover, the inherent diversity of herbal compounds can improve target engagement when properly formulated [4]. Therefore, Chinese medicinal formulas represent promising options for managing complex and chronic pediatric disorders such as constipation [5,6].

“YiNianJin” (YNJ) is a classic traditional Chinese medicine formula with over 400 years of history in pediatric clinical applications in China. It is widely used for children aged 0 to 6 years and has been shown to effectively promote digestion and relieve constipation [7]. In the context of pediatric constipation, which is primarily functional and associated with intestinal immaturity, YNJ is considered particularly suitable due to its synergistic regulation of intestinal motility, fluid balance, and inflammation. Modern pharmacological studies have demonstrated that the components of YNJ, such as rhubarb, morning glory seeds, areca nut, and ginseng, exert complementary effects on colonic mucus secretion, aquaporin expression, gut microbiota modulation, and anti-inflammatory responses. These findings provide a scientific basis for the traditional use of YNJ in relieving constipation in children and support its potential as a multitarget therapeutic agent for pediatric GI disorders. This discrepancy arises because the etiology of childhood constipation is primarily functional and related to intestinal immaturity, whereas in adults, the causes are more heterogeneous. YNJ is composed of 5.66% cinnabar (HgS), 18.87% rhubarb, 37.74% stir-fried morning glory seeds, 18.87% areca nut, and 18.87% ginseng, rendering it a well-established pediatric formula for constipation relief. Rhubarb alleviates constipation by stimulating colonic mucus secretion and increasing the expression of mucin 2 (MUC2) [8]. Morning glory seed exerts laxative effects through down-regulating aquaporin 3 (AQP3) expression and up-regulating AQP1 by promoting the abundance of *Lactobacilli*, particularly in children [9]. In addition, both areca nut and ginseng exhibit anti-inflammatory properties by reducing pro-inflammatory cytokines such as interleukin-1β (IL-1β), IL-6, and tumor necrosis factor-α (TNF-α) [10,11], thereby alleviating intestinal inflammation and mitigating intestinal wall damage.

However, the current oral administration of YNJ faces notable limitations [12], including GI irritation and the risk of Hg^2+^ accumulation in organs and blood [13]. These risks are particularly pronounced in children, whose immature organs and systems are more susceptible to toxic side effects. Additionally, the poor bioavailability of other active herbal components in YNJ further limits its overall therapeutic efficacy. These challenges collectively limit the clinical applications of YNJ.

The transdermal drug delivery system (TDDS) offers a promising alternative by avoiding first-pass metabolism [14,15], providing improved pharmacokinetic stability compared to oral administration, and reducing systemic exposure in the blood and organs [16]. By altering the route of administration, transdermal preparations can mitigate toxicity risks and enhance drug safety, thereby underscoring their clinical value [17]. However, the transdermal delivery of Chinese medicinal formula remains scarce. Herein, we propose a novel strategy of delivering YNJ transdermally to simultaneously reduce toxicity and enhance therapeutic efficacy. Nevertheless, the skin barrier function poses a challenge for transdermal delivery. Overcoming the challenges of low cutaneous permeability and insufficient tissue penetration of many natural compounds that are poorly soluble, highly polar, and/or potentially toxic (such as cinnabar) is essential to ensure the pharmaceutical effects of these compounds in vivo. To address these issues, we firstly designed and prepared the cinnabar into lipid vesicles, and intestinal wall penetrating peptides (TAT and CSK) was added to make it easier to reach the intestine, which is more conducive to the treatment of intestinal diseases. This strategy enhances skin permeation through lipid fusion with the stratum corneum, disruption of skin structure, and endocytosis-mediated uptake, while also providing sustained release and reducing toxicity by minimizing direct compound exposure.

**Fig. 1.**
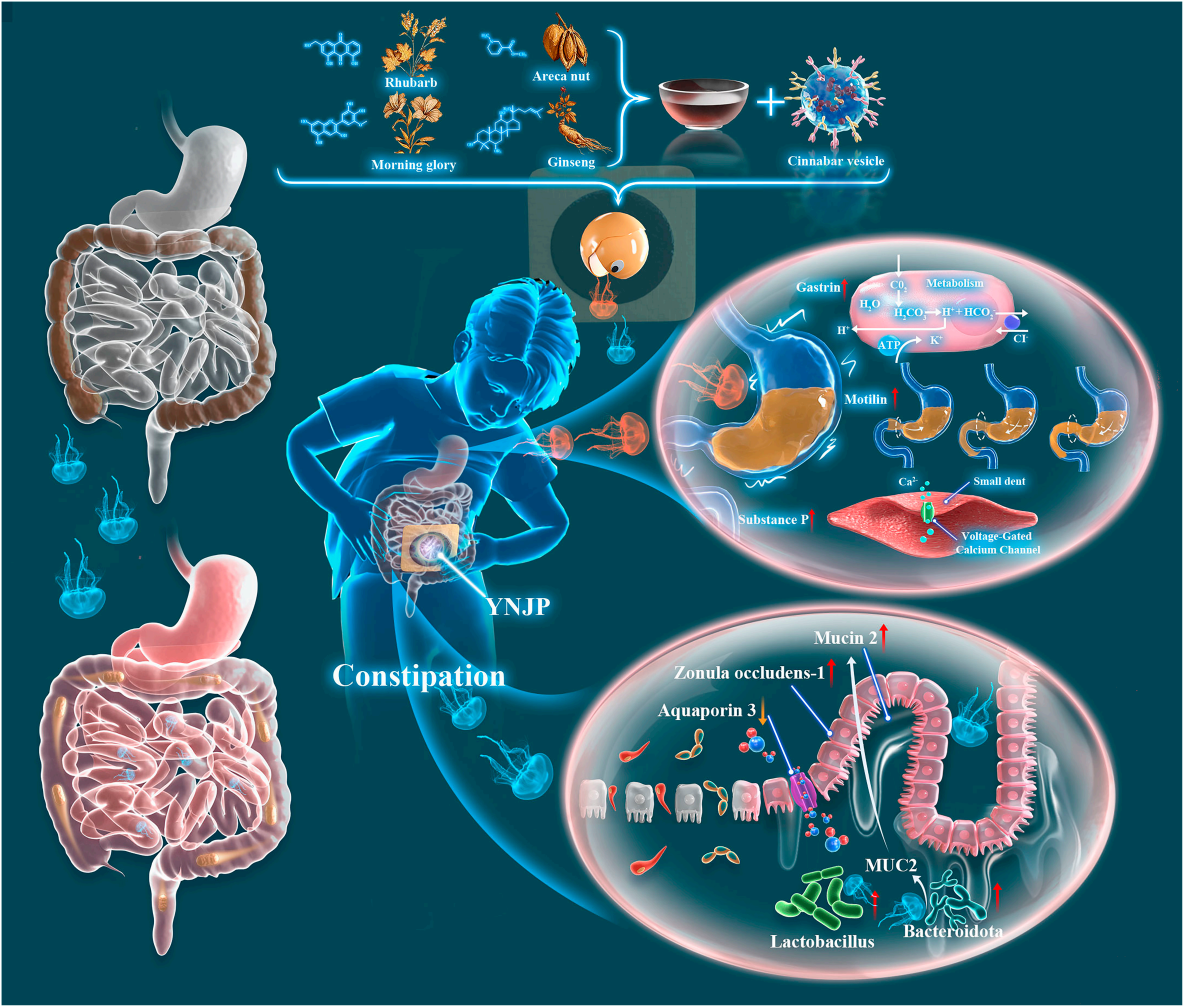
The areca nut, stir-fried morning glory seeds, rhubarb, and ginseng released from YNJP work synergistically to enhance the secretion of endocrine homeostatic transmitters; regulate mucin, aquaporin, and tight junction protein expression; and alleviate constipation by modulating intestinal metabolic homeostasis effects.

Moreover, since YNJ contains active ingredients derived from both plant and mineral sources with diverse physicochemical properties, the design of the transdermal matrix requires careful consideration. The matrix must be precisely tailored to accommodate the distinct characteristics of each type of ingredient. To address the challenges associated with the administration of YNJ, we developed a hybrid patch system (YNJ patches [YNJPs]), which incorporates a stromal skeleton patch engineered for the controlled release of both multiple active botanical ingredients and cinnabar vesicles (CVs). We further optimized the formulation of the vesicle-composite skeleton matrix. This system is combined with acupoint application technology, which is particularly suitable for treating constipation. The selected application site “Shenque point” (CV8, located at the navel area) features thinner skin, allowing for better penetration, higher absorption capacity, and a reduced first-pass effect. Moreover, drug absorption occurs directly at the CV8, which helps regulate GI tissues, improves targeting, and enhances therapeutic efficacy of GI constipation. This innovative strategy combines modern transdermal technology with traditional acupoint therapy to enhance transdermal efficiency and abdominal targeting, while reducing systemic toxicity. It offers a novel approach for the transdermal acupoint delivery of Chinese medicinal formulations in pediatric diseases and provides valuable insights into the development of delivery platforms that effectively balance safety and therapeutic efficacy (Fig. 1).

## Materials and Methods

### Materials and animals

Rhubarb, stir-fried morning glory seeds, areca nut, ginseng, and cinnabar were purchased from Shanghai Yuanye Biotechnology Co., Ltd. Polyoxyl (40) stearate and cholesterol were bought from Wuhan Rat Laibao Biotechnology Co., Ltd. The Cell Counting Kit-8 (CCK-8) was bought from Hangzhou Mingte Co., Ltd. The apoptosis and ROS kit was obtained from Shanghai Beyotime Biotechnology. Trizol reagent was bought from Shanghai Beyotime Biotechnology Co., Ltd. The TransScript One-Step gDNA Removal and cDNA Synthesis Mix kit was purchased from Suzhou Novoprotein Technology Co., Ltd. Loperamide (EPL) was purchased from Sigma-Aldrich (USA). The substance P (SP), motilin (MTL), and gastrin (GAS) kits were purchased from Shanghai Jianglai Biotechnology Co., Ltd. The primary antibodies of AQP3, tight junction protein 1 (ZO-1), and MUC2, and secondary antibodies were bought from Abcam Plc (USA).

Female C57BL/6 mice (20 ± 3 g) and female Sprague–Dawley (SD) mice (100 ± 10 g) were provided by Shanghai Slac Laboratory Animal Co., Ltd. (China). The feeding conditions for mice are a relative temperature of 18 to 22 °C, a relative humidity of 50% to 60%, and a light cycle of alternating brightness and darkness of 12/12 h. All animal experiments conducted adhered strictly to the guidelines set by the Experimental Animal Welfare and Ethics Committee of Zhejiang University and were approved by the relevant animal care and experimental procedures authorities.

### Preparation of CVs and YNJP

#### Preparation of CVs

Based on univariate analysis, the optimal combination of CVs was determined to be 1.5 g of polyoxyl 40 and 1.5 g of cholesterol, which were dissolved in 32 ml of ethanol to form a thin film by spin evaporation at 50 °C. Thirty milliliters of phosphate-buffered saline (PBS; containing 0.6 g cinnabar) was added, and the mixture was magnetically stirred for 15 min. Drug/lipid ratio is 1:6. Then, cell-penetrating peptide (TAT) and goblet cell-targeting peptide (CSK) (mass ratio is 1:3) are added. The mixture is shaken for 2 h and stored in a refrigerator at 4 °C. Finally, their stability in terms of time, light, and temperature was evaluated.

#### Preparation of YNJP

Firstly, 1 g of rhubarb, 1 g of areca nut, 1 g of ginseng, 2 g of morning glory, and 2 ml of CVs (containing 0.3 g of cinnabar) were crushed. CMC-Na (0.2 g) was dissolved with 5 ml of water as phase A. Then, 4.5 g of glycerol, 0.05 g of aluminum, 0.5 g of NP700, and 0.3 g of carbomer 940 were mixed evenly, and then each herb and CVs were added and mixed sustainably as phase B. Then, 0.05 g of tartaric acid was dissolved with 2 ml of water as phase C. Phase A was added to phases B and C successively. Moreover, polyvinylpyrrolidone (PVP K30, thickening agent) and kaolin were added as drug screening material. Finally, 1% azone was added as a penetration enhancer, stirred for 20 min, and was placed for 24 h. Finally, a YNJ controlled release patch (YNJP) was obtained. Finally, their stability in terms of shedding time, melt temperature, and degradation time was evaluated.

In subsequent cell and animal experiments, the dosages of YNJ and YNJP were calculated based on the total effective concentrations of rhubarb, areca nut, ginseng, morning glory, and CVs.

### Characterization of YNJP

#### TEM observation

The morphology of blank patches (P) and YNJPs was examined using transmission electron microscopy (TEM, Tecnai G2 Spirit, Japan).

#### Release of drugs in YNJP

The in vitro release profiles of YNJ and YNJP were studied using a Franz diffusion cell system (LOGAN, USA). A volume of 500 μl of YNJ (concentration of drug powder: 100 mg/ml)- and YNJP (concentration of drug powder: 100 mg/ml)-loaded CV were added to the donor compartment of the Franz diffusion cell, with a cellulose membrane (molecular weight, 12,000) sandwiched between the donor and receptor chambers. The receptor was filled with 15 ml of PBS. The receptor solution was stirred at 300 rpm using a magnetic bar, and the temperature was maintained at 35 ± 2 °C. Aliquots of 1 ml were withdrawn at 2, 12, 24, 48, and 72 h. Finally, the samples were detected using high-performance liquid chromatography (HPLC) and inductively coupled plasma (ICP) spectroscopy.

#### Skin penetration of drugs in YNJP

For the in vitro skin penetration test, Franz diffusion cells were used. Skin from SD rats was cut and mounted on the receptor compartment of the diffusion cell. Five hundred microliters of YNJ (concentration of drug powder: 100 mg/ml)- and YNJP (concentration of drug powder: 100 mg/ml)-loaded CVs was added to the donor compartment of the Franz diffusion cell, respectively. The receptor solution was stirred at 300 rpm using a magnetic bar, and the temperature was maintained at 37 ± 0.5 °C, and 1-ml aliquots were withdrawn at 0.5, 1, 2, 4, 8, 12, 24, 36, 48, and 72 h. Finally, the samples were detected using HPLC and ICP.

### HPLC detection

#### The detection of ginsenoside

Ginsenoside Rg1 was placed in a Soxhlet extraction apparatus (Shanghai Leao Test Instrument Co., Ltd), trichloromethane was added, and the mixture was heated and refluxed for 3 h. The trichloromethane solution was discarded, and the residue solvent was evaporated. The residue, along with a filter paper tube, was transferred into a 100-ml conical flask. n-Butanol saturated (50 ml) was precisely added with water, ultrasonically treated (power 250 W, frequency 50 kHz) for 30 min, and filtered. The initial filtrate was discarded; 25 ml of the secondary filtrate was accurately measured and placed in an evaporating dish to dry; the residue was dissolved in methanol, transferred to a 5-ml volumetric flask, diluted to the mark with methanol, and shaken evenly; and the secondary filtrate was taken.

#### The detection of pharbitin

Pharbitin and 25 ml of methanol were precisely added into a conical flask with a stopper, weighed, heated, and refluxed for 1 h. Then, it was cooled down, weighed again, and shaken. The filtrate (5 ml) was accurately measured and placed in a flask, and the solvent was evaporated. Then, 10 ml of 8% hydrochloric acid solution was added and ultrasonically treated for 2 min, and 10 ml of chloroform was added. The mixture was then heated and refluxed for 1 h, cooled, and placed in a separatory funnel. The container was washed with a small amount of chloroform and poured into the separatory funnel to separate the chloroform layer. The acid solution was extracted 3 times with chloroform (10 ml each time). All chloroform solution was combined, the solvent was recovered under reduced pressure, and methanol was added to dissolve the residue. The solution was transferred to a 10-ml volumetric flask. Finally, the methanol was added to the mark, shaken evenly, and filtered, and the filtrate was taken.

#### The detection of total anthraquinones

Total anthraquinones were placed in a 100-ml conical flask with a stopper. Methanol (20 ml) was precisely added, weighed, ultrasonically treated for 1 h (power 250 W, frequency 40 kHz), cooled, weighed again, and shaken.

The detection was carried out using a C_18_ chromatographic column (Agilent, USA), with acetonitrile and water as the mobile phases. Gradient elution was carried out using acetonitrile as mobile phase A and water as mobile phase B as stipulated in Table S2. The detection wavelength was 203 nm. The column temperature was set to 37 °C, the flow rate was 0.3 ml/min, and the injection volume was 10 μl.

#### The detection of arecoline

The liquid was taken and placed in a conical flask with a stopper. Ether (50 ml) was added. Three milliliters of carbonate buffer solution (including 0.64 g/ml sodium carbonate and 0.19 g/ml sodium bicarbonate) was dissolved in water to 100 ml. They were stood for 30 min, shaken, heated, and refluxed for 30 min. Then, the ether solution was separated and added to an evaporating dish containing 1 ml of phosphoric acid solution (5→1,000). The residue was heated and refluxed with ether for extraction twice (30 ml and 20 ml), each time for 15 min. The ether solutions were combined and placed in the same evaporating dish, and the ether was removed. The residue was dissolved in 50% acetonitrile solution and transferred to a 25-ml volumetric flask. Acetonitrile (50%) was added to the mark, shaken evenly, and filtered. Finally, the filtrate was taken. The detection was carried out using a strong cation exchange (SCX)-strong positive exchange resin chromatographic column (Agilent, USA). The mobile phase was acetonitrile–phosphoric acid solution (2→1,000, pH adjusted to 3.8 with concentrated ammonia test solution) (55: 45). The detection wavelength was 215 nm. The temperature was set to 37 °C, the flow rate was 0.3 ml/min, and the injection volume was 10 μl.

### ICP detection

The concentration of Hg^2+^ in the solution was detected by ICP spectroscopy (PerkinElmer, USA). One milliliter of supernatant or suspension was placed into a clean PTFE digestion tube. Hydrochloric acid (2 ml) and 6 ml of nitric acid were gradually added, and the digestion tube was placed inside a microwave digestion oven. Follow the digestion procedure outlined in Table S3. Once digestion is complete, the solution was cooled to room temperature. The digested solution was transferred from the tube into a 50-ml volumetric flask and diluted with ultrapure water up to the mark. To prepare the Hg^2+^ test solution, this mixture was diluted by a factor of 100. The samples were prepared for ICP detection after digestion, as described in Table S4.

### In vitro experiment

#### Cell viability assay

The CCK-8 assay was used to assess the effect of YNJP on the proliferation of interstitial cells of Cajal (ICCs). Initially, ICCs were cultured in dishes until they reached 90% confluence in the iCell primary stromal cell culture system (iCell Bioscience Inc, Shanghai) supplemented with 10% fetal bovine serum. Moreover, the cells were harvested and seeded into a 96-well plate at a density of 100 μl per well. ICC cells were divided into a blank group, a model group and a treated group. After the 24 h of incubation, the cells were treated with different concentrations of YNJ (0 to 100 μg/ml), YNJP (0 to 100 μg/ml), and EPL (0 to 1,000 μg/ml) for 24 h. Moreover, another experiment involved treating with 200 μg/ml EPL for 24 h. After successful modeling, 50 μg/ml YNJ and 50 μg/ml YNJP were added for 24 h, respectively. Then, remove the original culture medium, 10 μl of CCK-8 solution was added to each well, and the cells were further incubated at 37 °C for 4 h without light. Subsequently, the absorbance was measured at 570 nm using an infinite F50 absorbance microplate reader (TECAN, Austria).

Human immortalized epidermal (HaCaT) or human skin fibroblast (HSF) cells were seeded in 96-well plates at a density of 5,000 cells/well and allowed to adhere for 24 h. The cells were then treated with varying concentrations of YNJP (0, 10, 20, 30, 40, and 50 μg/ml) for 24 h. Following treatment, the culture medium was carefully replaced with serum-free Dulbecco's Modified Eagle Medium (DMEM) containing 10% CCK-8 reagent, and the cells were incubated for an additional 4 h. Absorbance was measured at 450 nm using an ELISA microplate reader (Infinite F50, TECAN, Austria).

#### Flow cytometry

The effects of YNJ and YNJP on apoptosis in ICCs were evaluated using a flow cytometric assay. Initially, ICC cells were incubated in 75-cm^2^ culture dishes and subsequently seeded into 6-well plates. These cells were then cultured at 37 °C for 24 h, and the cells were treated with EPL (200 μg/ml), YNJ (50 μg/ml), and YNJP (50 μg/ml), respectively, at 37 °C for an additional 24 h. Both blank and treated groups of ICC cells were harvested using trypsin digestion and centrifuged at 1,000 *g* for 5 min. After washing the cells 3 times with PBS at 4 °C, they were stained with fluorescein isothiocyanate 7-aminoactinomycin D and P-phycoerythrin for 15 min.

#### Detection of ROS

The effects of YNJ and YNJP on the release of reactive oxygen species (ROS) in ICC cells were investigated using a flow cytometric assay. The cells were cultured in 6-well plates and treated with EPL (200 μg/ml), YNJ (50 μg/ml), and YNJP (50 μg/ml), respectively, at 37 °C for 24 h. After treatment, the cells were washed 3 times with PBS and stained with 10 μM dichlorodihydrofluorescein diacetate (DCFH-DA). The fluorescence intensity of the cells was measured via flow cytometry after 20 min.

#### Detection of endocrine homeostatic transmitters and oxidative stress levels

ICC cells treated with EPL (200 μg/ml), YNJ (50 μg/ml), and YNJP (50 μg/ml) were digested with trypsin and centrifuged at 1,000 rpm for 5 min. The supernatant was collected, and each endocrine homeostatic transmitter indicator, including SP, MTL, and GAS, and oxidative stress markers, including superoxide dismutase (SOD), glutathione (GSH), lactate dehydrogenase (LDH), and malondialdehyde (MDA), were measured using ELISA kits according to the manufacturer’s instructions.

### In vivo experiments

#### Modeling and administration of mice

EPL suspension (3 mg/kg in PBS) was administrated by intraperitoneal injection once daily from day 1 to day 10 to induce constipation. Moreover, 5.0, 6.0, 7.0, and 8.0 g/kg/day groups were set up, and eventually found that in the YNJ-or group or YNJP-tr group, all indicators were normal at the 6.3 g/kg/day dose. Therefore, the blank group was administrated with the same dosage of PBS following the same schedule. YNJ/YNJP/DGE suspension (6.3 g/kg in PBS) was administrated once daily from day 10 to day 20. Among them, YNJ is oral delivery (YNJ-or), and YNJP (YNJP-tr) and DGE (DGE-tr) are transdermal delivery. The water intake and food intake of mice were detected on day 15 and day 20, respectively. The mice in all groups fasted for 16 h on the 20th day, tissue (stomach, small, and large intestine), and fecal samples were collected for analysis. All collected samples were stored at −80 °C.

#### Detection of the intestinal propulsion rate

Mice were fasted overnight (16 h) to empty their intestinal contents. Mice were orally fed 0.25 ml of activated charcoal suspension and then sacrificed under anesthesia after 20 min. The abdominal cavity of mice was quickly opened, and the entire intestine from the pylorus to anus was dissected. The intestinal propulsion rate was calculated as *D* = L2/L1×100%, L1 was the full length of the intestine after straightening without tension, and L2 was the movement length of activated carbon in the intestine.

#### Detection of the fecal water content

The mice were placed in a clean cage next day, and before the administration of the treatment, the feces were collected and weighed, and the wet weight of feces was recorded as W1. The collected feces were paced in an oven for drying and dehydration at 105 °C, and the dry weight was recorded as W2. Finally, the water content of fecal was calculated as *R* = (W1 − W2)/W1 × 100%.

### Detection of endocrine homeostatic transmitters levels

The stomachs of mice were removed, triturated, and centrifuged, and the supernatant is taken. The levels of endocrine homeostatic transmitters, including SP, MTL, and GAS, were tested according to the instructions provided with the respective ELISA kits.

### Immunofluorescence detection

Intestine tissues were fixed with 4% paraformaldehyde for 48 h, dehydrated by gradient ethanol, embedded in paraffin, and sectioned into 10-μm slices. Sections were baked, dewaxed, boiled in antigen repair solution for 20 min, and sealed at room temperature for 20 min with goat serum sealing solution. The primary antibodies (1:5,000) of AQP3, ZO-1, and MUC2 were added, incubated overnight at 4 °C, and washed with PBST. Fluorescent secondary antibody (1: 500) was incubated at room temperature for 60 min, washed 3 times in PBST (5 min), stained with DAPI (4′,6-diamidino-2-phenylindole) for 10 min, and washed with PBST. Tablets were sealed by anti-fluorescence quenching and observed using a fluorescence microscope, and results were quantified using ImageJ software.

### 16S rRNA analysis

Fecal samples from the blank, model, DGE, and YNJ groups were subjected to 16S rRNA sequencing. DNA was extracted from feces and amplified by PCR, and PCR products were then aliquoted and purified. The constructed library was quantified by Qubit and Q-PCR, and the PE250 was sequenced using NovaSeq6000. The raw data obtained from sequencing were spliced and filtered to obtain clean data. Based on the clean data, the final ASVs were obtained by denoising the clean data through DADA2 and used for further analysis.

### Quantitative real-time PCR

Total RNA isolation from intestines were performed using Trizol reagent, followed by cDNA synthesis using the TransScript One-Step gDNA Removal and cDNA Synthesis Mix kit. Quantitative analysis of inflammation-related mRNA expression was conducted via real-time PCR (RT-qPCR, Thermo Fisher Scientific, USA) using β-actin as the endogenous control. Gene-specific primers for MUC 2 and β-actin (sequences listed in Table S5) were designed using NCBI and SnapGene software. The relative expression levels of target genes were quantified using the 2^−ΔΔCt^ method.

### Gas chromatography–mass spectrometry detection of SCFAs

#### Preparation of standard solutions

Short-chain fatty acid (SCFA) standard samples were accurately weighed, dissolved in ultrapure water, diluted to volume, and prepared using single standard solutions. Appropriate volume of single standard solution was taken and diluted with ultrapure water to obtain a mixed standard stock solution, where the mass concentration of acetic acid is 5.0 mg/ml, the mass concentration of propionic acid is 2.5 mg/ml, and the mass concentration of butyric acid is 0.5 mg/ml. Before the experiment, ultrapure water was used to prepare a series of concentration mixed standard solutions with a gradient of 4 to 2,000 times.

#### Solid-phase extraction conditions of intestine

The intestinal tissue was taken, ground, and extracted with a 100-μm solid-phase microextraction head. The sample was incubated at 40 °C, stirred at 1,000 rpm, incubated for 20 min, extracted at 40 °C for 30 min, and analyzed for 5 min.

#### Gas chromatography conditions

The chromatographic column is Agilent HP-INNOVAX (30 mm × 0.32 mm, 0.25 μm). The carrier gas is helium gas (purity of 99.999%), with a flow rate of 1.0 ml/min, injection in non-split mode, an inlet temperature of 250 °C, and a transmission line temperature of 280 °C. Heating program: The initial temperature is maintained at 60 °C for 2 min, then raised to 200 °C at a rate of 10 °C/min for 5 min, and finally raised to 250 °C at a rate of 10 °C/min for 2 min.

#### Mass spectrometry conditions

Electron bombardment ion source, ion source temperature 230 °C, electron energy 70 eV, quadrupole temperature 150 °C, selective ion scanning, solvent delay 5 min, and selected ion parameters are as shown in Table S6.

### Hematoxylin–eosin staining

The intestinal tissues of mice were collected, paraffin-embedded, and sectioned at 3 to 6 μm thickness. Sections were stained with hematoxylin–eosin (H&E), then scanned with an orthogonal fluorescence microscope equipped with a camera (Leica Ltd.).

### Statistical analysis

All values are expressed as mean ± SD, *n* = 3. Statistical significance was determined using Student *t* test. For multiple comparisons, one-way analysis of variance followed by Tukey’s multiple comparisons test was performed. A *P* value < 0.05 was considered statistically significant. GraphPad prism 9.0 was used for data analysis and figure generation.

## Results

### Preparation and optimization of CVs

**Fig. 2.**
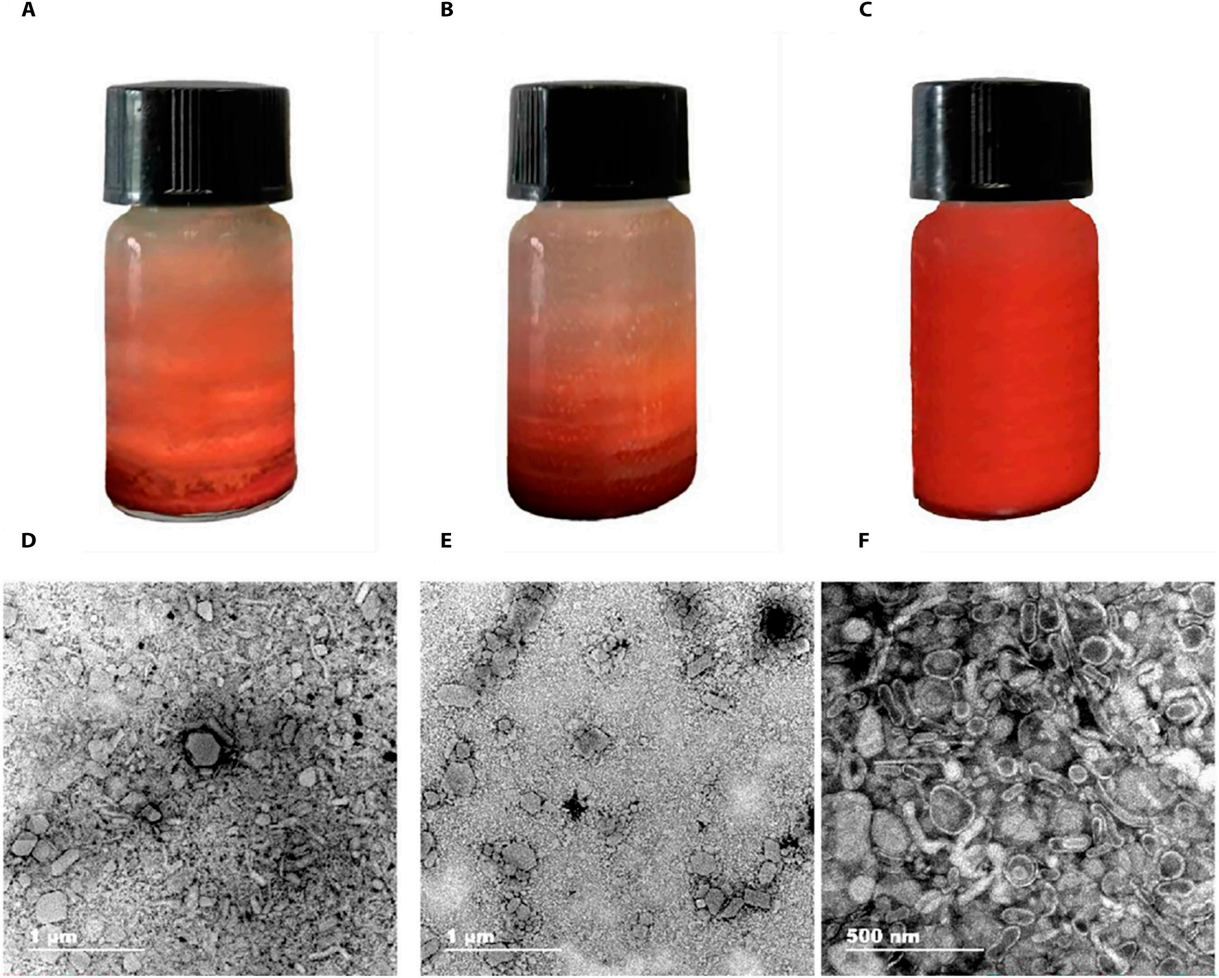
Characterization of CVs. (A to C) Macroscopic morphology observation of cinnabar vesicles (CVs) prepared by different preparation methods. (D to F) Transmission electron microscopy observation of CVs prepared by different preparation methods.

**Fig. 3.**
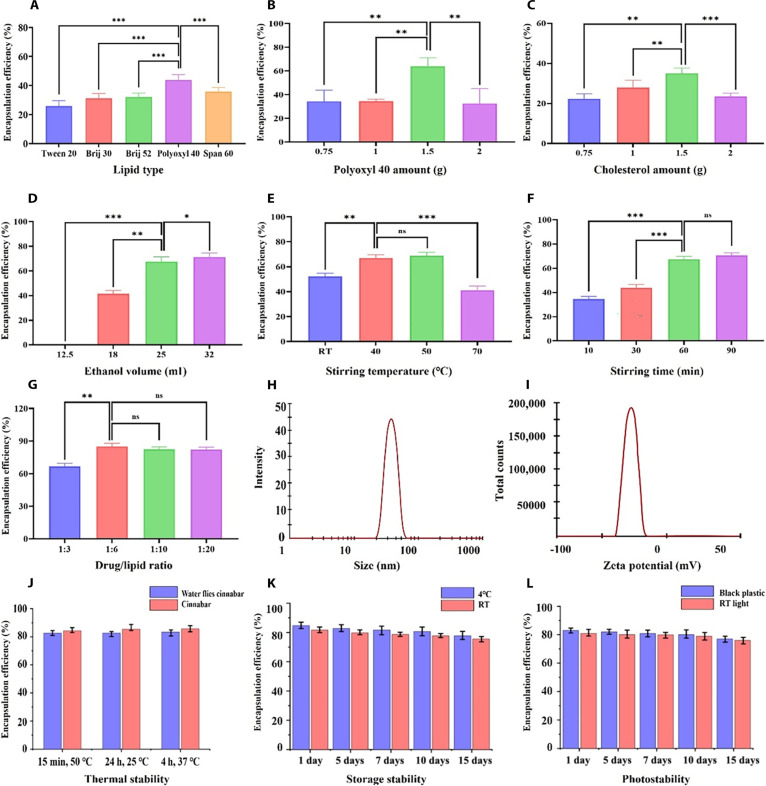
Univariate analysis for the preparation and optimization of cinnabar vesicles (CVs). Investigation for (A) lipid type, (B) polyoxyl 40 amount, (C) cholesterol amount, (D) ethanol volume, (E) stirring temperature, (F) stirring time, and (G) drug-to-lipid ratio. (H) The particle size and (I) potential of cinnabar vesicles. (J to L) Stability assessment of cinnabar vesicles, included thermal stability, storage stability, and photostability. ^ns^*P* > 0.05, *P < 0.05, **P < 0.01, and ***P < 0.001.

Firstly, CVs were initially prepared using the injection method, in which a cinnabar solution (in PBS) was added into an ethanol solution under stirring. However, this approach resulted in cinnabar precipitation and poor miscibility with ethanol (Fig. 2A and D). Therefore, the rotary evaporation method was adopted instead. A univariate analysis was employed to screen key formulation parameters, including the type of lipid (Fig. 3A), the amount of polyoxyl 40 (Fig. 3B), the amount of cholesterol (Fig. 3C), the volume of ethanol (Fig. 3D), stirring temperature (Fig. 3E), stirring time (Fig. 3F), and the drug-to-lipid ratio (Fig. 3G). The encapsulation rate was used as the indicator for screening and validation. Based on the univariate analysis results, the optimal formulation and process parameters for CVs were determined as follows: 1.5 g of polyoxyl 40, 1.5 g of cholesterol, and 32 ml of ethanol were used to form a thin film by rotary evaporation at 50 °C. Then, 30 ml of PBS containing 0.6 g of cinnabar was added, and the mixture was magnetically stirred at 50 °C for 15 min with a drug-to-lipid ratio of 1:6. Then, cell-penetrating peptide (TAT) and goblet cell-targeting peptide (CSK) (mass ratio is 1:3) are added. The mixture is shaken for 2 h, and the CVs are prepared. Furthermore, compared to vesicles prepared with span 60 (Fig. 2B and E), those formulated with polyoxyl 40 exhibited the best appearance and morphology, with a uniform mixture (Fig. 2C and F).

The particle size and zeta potential of CVs were analyzed using a laser particle size analyzer. The vesicles exhibited a size distribution from 50 to 100 nm (Fig. 3H), while the potential data are presented in Fig. 3I. Furthermore, the thermal, storage, and photostability of the CVs were evaluated. The results demonstrated that the thermal stability of CVs was superior to that of cinnabar water-dispersible formulations, suggesting that vesicle encapsulation can enhance the thermal stability of cinnabar (Fig. 3J). After being stored at room temperature (25 °C) for 7 d, obvious stratification was observed in the CVs, whereas samples stored at 4 °C exhibited only slight stratification after 10 d. These findings indicate that CVs demonstrate improved storage stability under refrigerated conditions at 4 °C (Fig. 3K). Photostability analysis revealed a time-dependent decline in encapsulation efficiency under both light and dark conditions. However, the degradation was slower under light-protected conditions (Fig. 3L).

### Preparation and characterization of YNJP

**Fig. 4.**
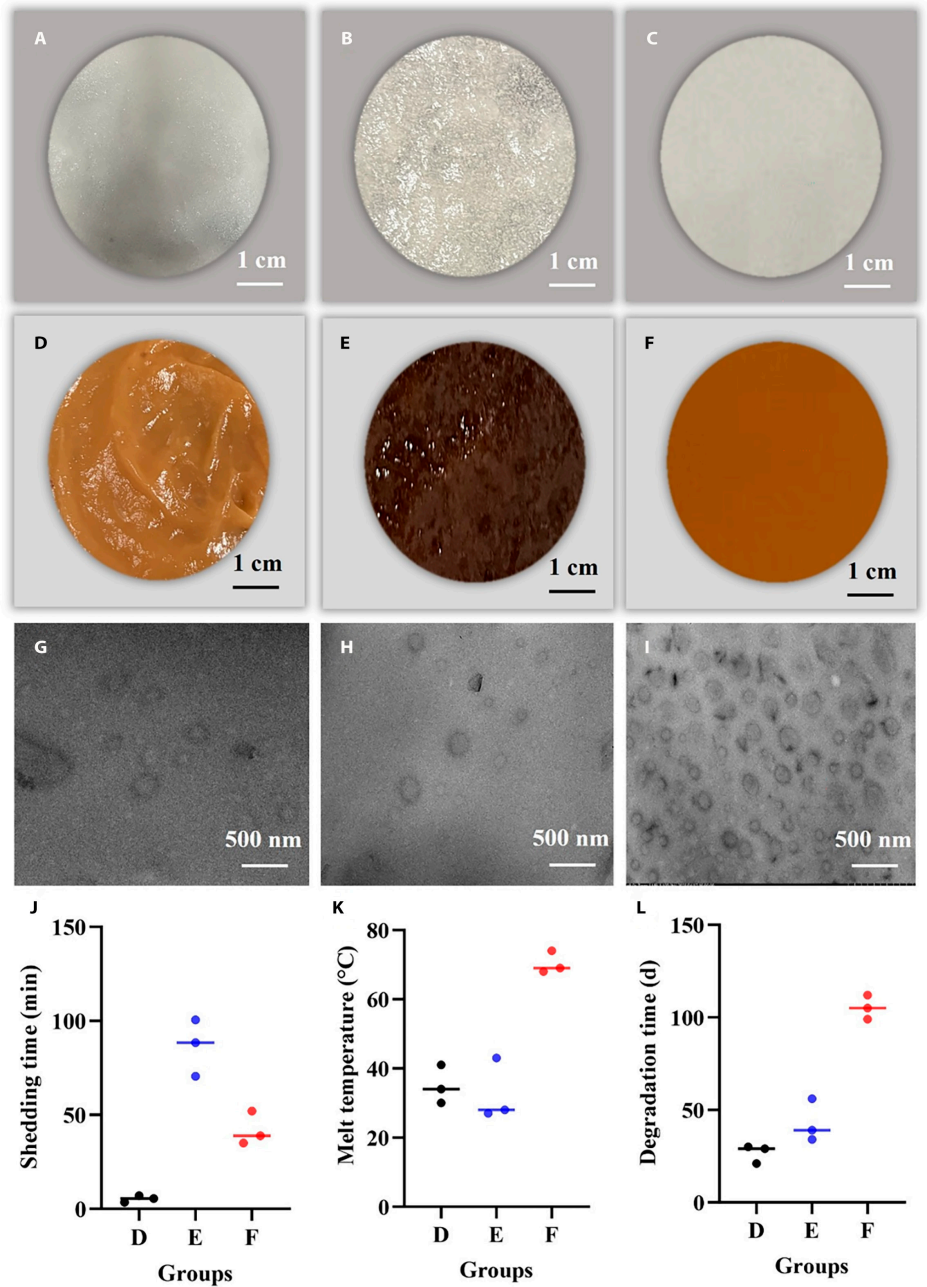
Characterization of YNJP. The macroscopic morphology of (A to C) blank patches (P), and (D to F) YNJ patch (YNJP) using different preparation methods; scale bar = 1 cm. (G to I) Transmission electron microscopy observation of YNJP using different preparation methods; scale bar = 500 nm. (J) Shedding time, (K) melt temperature, and (L) degradation time of YNJP.

Firstly, 0.2 g of CMC-Na was dissolved in 5 ml of water to form phase A. Then, 4.5 g of glycerol, 0.05 g of aluminum, 0.5 g of NP700, and 0.3 g of carbomer 940 were mixed uniformly. Subsequently, each herb and the CVs were added and mixed consistently to form phase B. Next, 0.05 g of tartaric acid was dissolved in 2 ml of water to create phase C. Phase A was successively added to phase B and then phase C. Finally, 1% azone, as a penetration enhancer, was incorporated into the mixture, and the resulting formulation was stirred for 20 min and allowed to stand for 24 h. The blank patch (P) prepared under these conditions exhibited a smooth texture (Fig. 4C). Subsequent attempts involved adding areca nut, stir-fried morning glory seeds, rhubarb, and ginseng for further evaluation. The resulting patch was delicate, evenly dispersed, and suitable for subsequent studies (Fig. 4F). Moreover, it has moderate shedding time (Fig. 4J), higher melt temperature (Fig. 4K), and longer degradation time (Fig. 4L). TEM analyses confirmed the smooth morphology of the patch, with CVs visibly adhered to the matrix while maintaining their structural integrity (Fig. 4I).

Moreover, the blank patch (P) matrix was prepared using PVP K30 as a thickening agent, which slightly increased the viscosity of the patch (Fig. 4A). After adding YNJ powder, the viscosity decreased, resulting in a noticeably watery consistency that reduced its adhesiveness to the skin (Fig. 4D). When kaolin was introduced, the patch viscosity decreased further and mixing became uneven (Fig. 4B). Subsequent addition of YNJ powder led to a more pronounced granular texture in the resulting patch (Fig. 4E), and it is too greasy (Fig. 4J). TEM observation of the patch with attached CVs showed a relatively low number of CVs with incomplete morphological structures (Fig. 4G and H).

### The release and skin penetration of drugs in YNJP

**Fig. 5.**
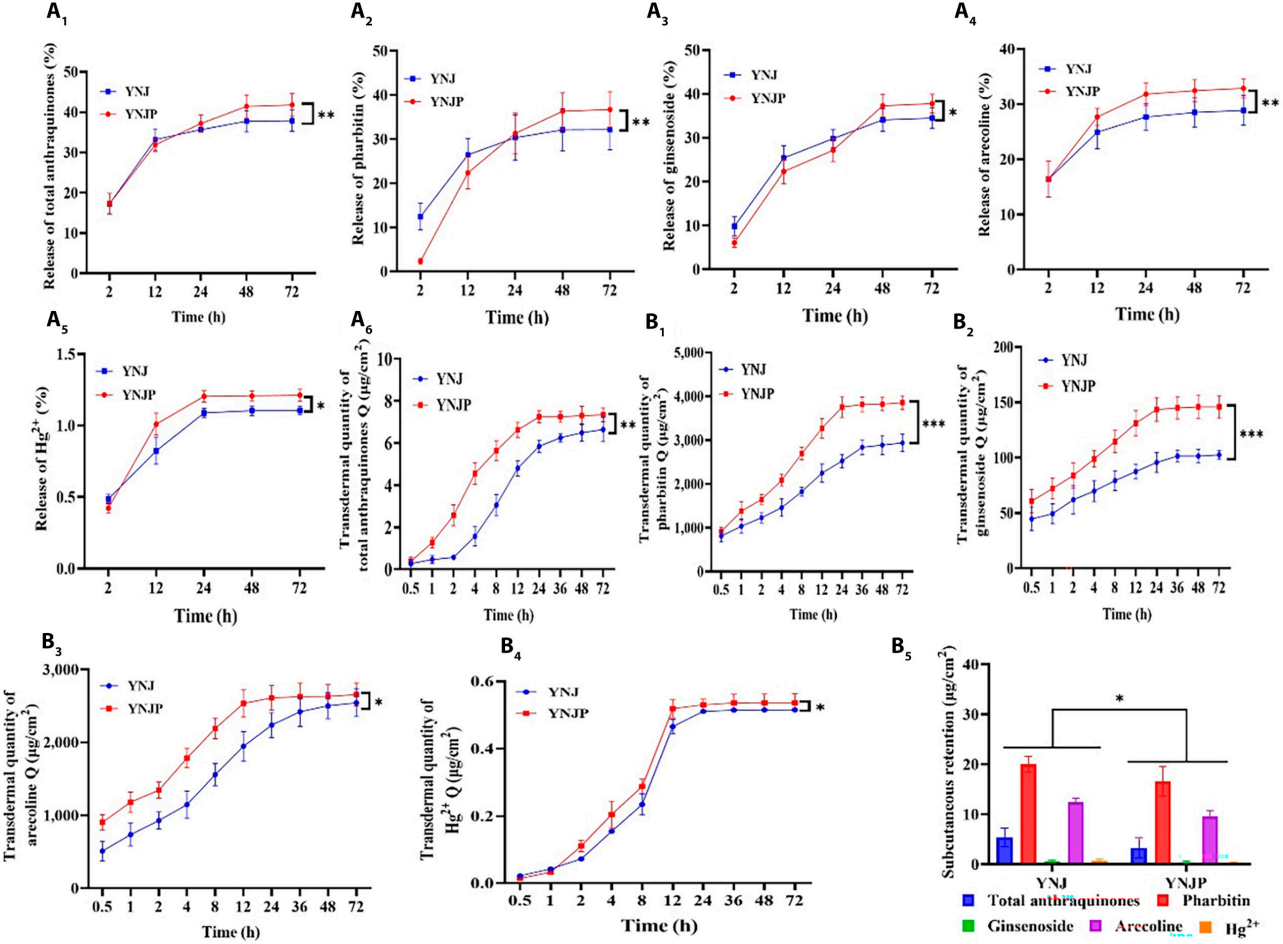
The release and skin penetration of drugs in YNJP. (A_1_ to A_6_) Comparison of in vitro release of total anthraquinones, pharbitin, ginsenoside, arecoline, and Hg^2+^ in the YNJ and YNJP. (B_1_ to B_4_) Comparison of in vitro skin penetration rate of total anthraquinones, pharbitin, ginsenoside, arecoline, and Hg^2+^ in the YNJ and YNJP. (B_5_) Subcutaneous drugs retention of YNJ and YNJP in vitro. *P < 0.05, **P < 0.01, and ***P < 0.001.

The YNJP was prepared using carboxymethyl cellulose sodium (CMC-Na) as the primary matrix material [18]. Our findings demonstrate that both drug release (Fig. 5A_1_ to A_5_) and skin penetration (Fig. 5B_1_ to B_5_) of YNJ and YNJP increased over time. Notably, the YNJP group exhibited sustained-release characteristics for 1 to 3 d. Over the 72-h period, the cumulative release of total anthraquinones, pharbitin, ginsenoside, arecoline, and Hg^2+^ from YNJP was significantly higher than that from conventional YNJ, with increases ranging from 1.12- to 1.43-fold (*P* < 0.05). Similarly, the cumulative transdermal penetration of these 5 components was markedly elevated relative to in the YNJP group compared to the YNJ group (*P* < 0.05). Furthermore, YNJP was associated with significantly lower intradermal drug retention (Fig. 5A_6_, *P* < 0.05), indicating enhanced transdermal delivery efficiency with reduced skin deposition. These results suggest that YNJP is a highly promising transdermal formulation, offering both enhanced drug release and prolonged therapeutic duration.

In addition, a critical mechanistic question in transdermal delivery of Chinese medicinal formulas is whether the active components can reach the intestinal target in sufficient quantities to exert therapeutic effects. To address this, we directly quantified the concentrations of 5 major active components, total anthraquinones, pharbitin, ginsenoside, arecoline, and Hg^2+^, in intestinal tissue following transdermal administration of YNJP. Remarkably, all 5 components achieved significantly higher intestinal concentrations in the YNJP-transdermal group compared with the oral YNJ group (Fig. S1). This finding provides direct in vivo evidence that the active constituents not only penetrate the skin barrier but also enter the systemic circulation and effectively accumulate at the intestinal target site. The superior intestinal targeting of YNJP is likely attributable to the combined effects of the transdermal route (bypassing GI degradation and first-pass metabolism) and the engineered nanovesicle system (CVs) modified with TAT and CSK peptides to enhance intestinal homing. In addition to the biodistribution data, pharmacokinetic profiling revealed that YNJP transdermal administration exhibits sustained-release characteristics (Fig. S2). These complementary data support the conclusion that YNJP not only delivers active components to the target organ but does so with enhanced efficiency compared to the traditional oral formulation.

### In vitro anti-constipation effect of YNJP by relieving intestinal cell damage

**Fig. 6.**
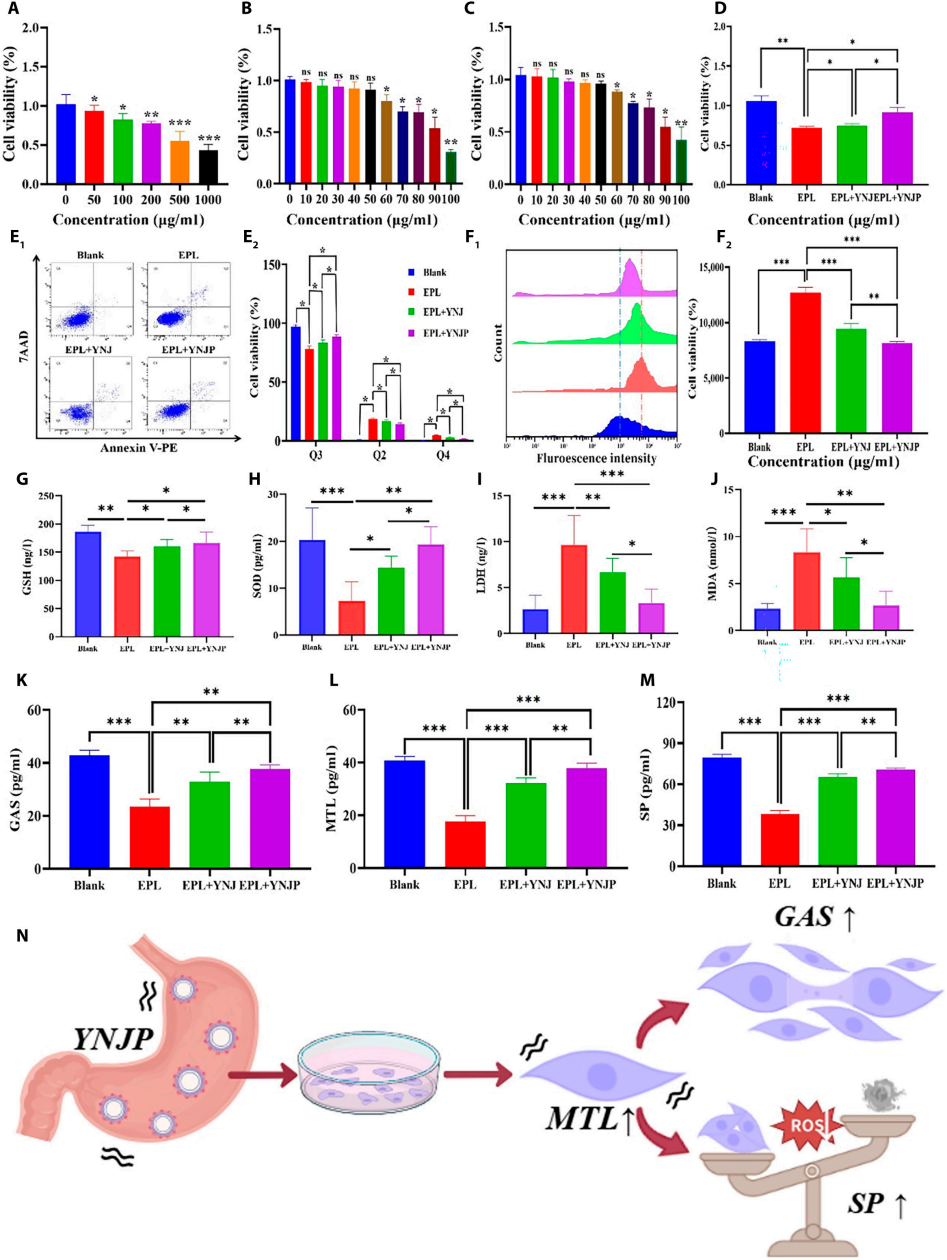
Anti-apoptotic and antioxidant effects of YNJP, along with its promotion of endocrine homeostatic transmitter production in vitro. (A) The ICC cell viability of EPL (0 to 1,000 μg/ml), (B) YNJ (0 to 100 μg/ml), and (C) YNJP (0 to 100 μg/ml). (D) Protective of ICC cells by YNJ/YNJP. The inhibitory effects of (E_1_ and E_2_) YNJ and YNJP on the apoptosis of ICC cells induced by EPL. The inhibitory effects of (F_1_ and F_2_) YNJ and YNJP on the ROS in ICC cells induced by EPL. The effects of YNJ and YNJP on the (G) GSH, (H) SOD, (I) LDH, and (J) MDA levels in EPL-induced ICC cells. The effects of YNJ and YNJP on the levels of endocrine homeostatic transmitters (K) GAS, (L) MTL, and (M) SP in ICC cells induced by EPL. (N) Mechanism diagram of YNJP’s anti-constipation effect in ICC cells. ^ns^*P* > 0.05, *P < 0.05, **P < 0.01, and ***P < 0.001.

Slow transit constipation (STC) is a prevalent GI disorder whose pathogenesis involves dysfunction of ICCs [19]. ICCs regulate intestinal motility through slow-wave generation and neuromuscular signaling [20]. Their depletion or abnormal distribution disrupts smooth muscle contraction, leading to STC. Opioid agents such as EPL are known to exacerbate STC by inhibiting peristalsis and causing GI cells damage [21]. In this study, an in vitro ICC injury model was established using EPL (200 μg/ml, Fig. 6A, *P* < 0.01). Treatment concentrations of 50 μg/ml for both YNJ and YNJP were selected (Fig. 6B and C, ^ns^*P* > 0.05), because the cell activity at this concentration is relatively better compared to other concentrations. Compared to YNJ, YNJP demonstrated a more pronounced protective effect on ICC cells in vitro (Fig. 6D, *P* < 0.05).

Given that constipation can induce oxidative stress and even apoptosis in ICCs [22], we further evaluated these pathological effects. Flow cytometry analysis revealed that both YNJ and YNJP (50 μg/ml) significantly attenuated EPL-induced apoptosis (Fig. 6E_1_ and E_2_, *P* < 0.05) and reduced ROS levels (Fig. 6F_1_ and F_2_, *P* < 0.01). They elevated antioxidant markers, GSH (Fig. 6G) and SOD (Fig. 6H), while reducing oxidative damage, LDH (Fig. 6I) and MDA (Fig. 6J). Furthermore, YNJ and YNJP increased the secretion of endocrine homeostatic transmitters, including GAS, MTL, and SP (Fig. 6K to M, *P* < 0.01), which are associated with anti-inflammatory effect, promoted GI cell proliferation, and enhanced GI motility and tissue repair (Fig. 6N) [23]. In summary, our findings indicate that YNJPs exert a dual effect of inhibiting inflammatory and alleviating constipation.

### In vivo anti-constipation effect of YNJP by stimulating GI motility

**Fig. 7.**
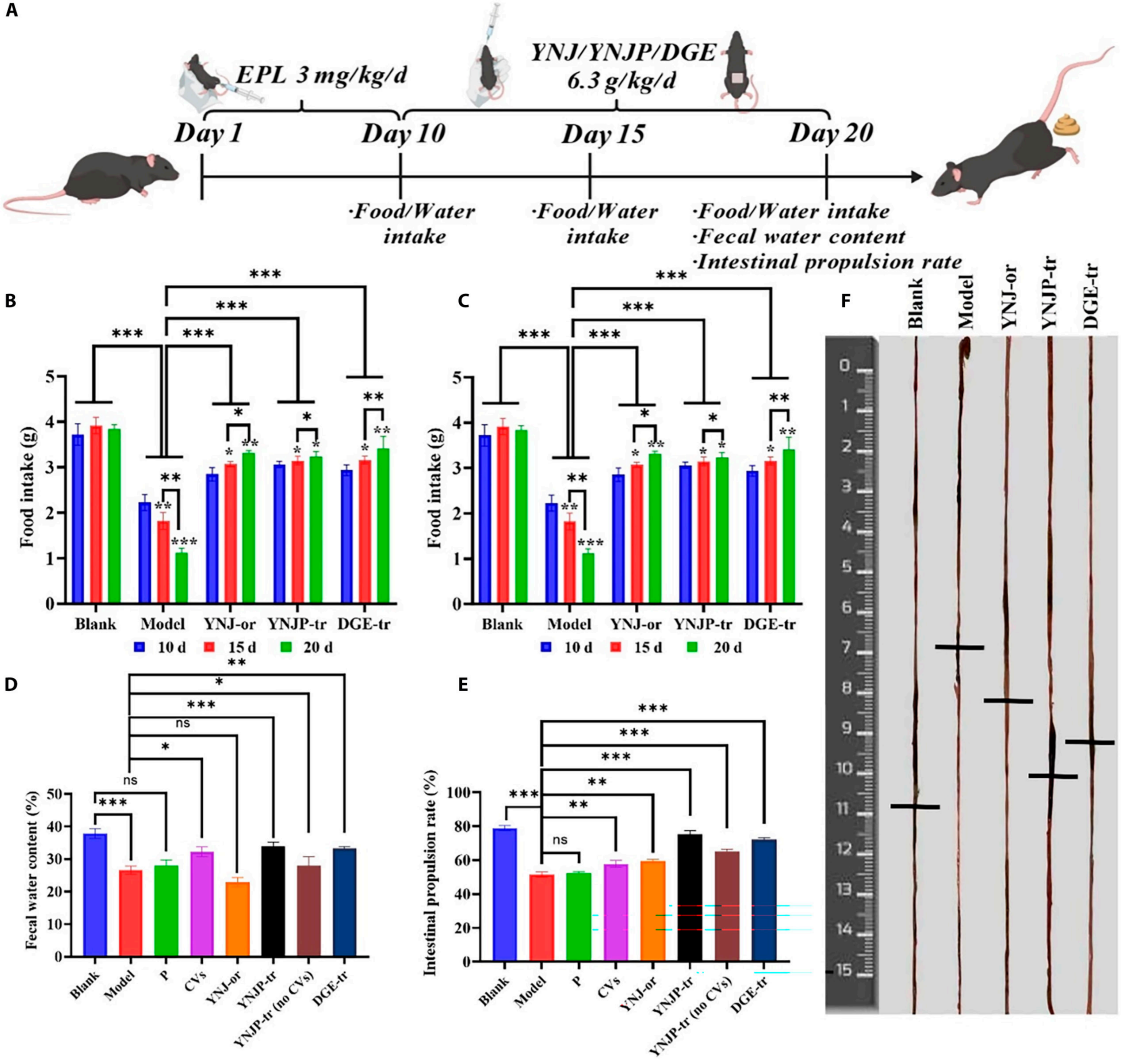
Effect of YNJP on gastrointestinal motility in a mouse model of EPL-induced. (A) Schematic of the modeling and drug administration process. (B) Food intake and (C) water intake of mice from 10 to 15 and 20 d. (D) Fecal water content in each group. (E) Intestinal propulsion rate in each group. (F) The graph of the small intestinal propulsion, including blank, model, blank patch (P), cinnabar vesicle (CVs), YNJ-or, YNJP-tr, YNJP-tr (no CVs), and DGE-tr. *P < 0.05, **P < 0.01, and ***P < 0.001.

STC is clinically characterized by infrequent bowel movement and straining during defecation, primarily resulting from impaired colonic motility [24]. The protective effects of YNJP against EPL-induced damage in ICCs were validated through in vitro assays. To further evaluate the therapeutic potential of YNJP in vivo, an STC mouse model was established via intraperitoneal injection of EPL (Fig. 7A). Food and water intake were monitored to assess the model. Significantly reduced food and water intake were observed in the model group compared to the blank group on days 15 and 20, which increased following YNJ-or (oral delivery, 6.3 g/kg/d) or YNJP-tr (transdermal delivery, 6.3 g/kg/d) treatment (Fig. 7B and C, *P* < 0.001), reaching levels comparable to the positive control group transdermal delivery treated with Ding Gui Er umbilical patch (DGE-tr), and the effect of YNJP-tr is better than YNJ-or (*P* < 0.001). DGE is a traditional Chinese herbal patch used to relieve intestinal discomforts such as diarrhea, abdominal pain, and bloating, particularly those caused by cold or indigestion in children. It functions by delivering warming herbs such as clove and cinnamon transdermally through the navel, thereby dispelling cold, regulating digestion, and relieving discomfort.

Constipation pathophysiology involves delayed intestinal motility and fecal stasis, leading to excessive water absorption and hardened, dry stools [25]. Thus, fecal water content and intestinal propulsion rate are critical parameters for assessing constipation severity. Experimental results showed that constipated mice developed significantly drier stools with reduced water content compared to the blank group (Fig. 7D, *P* < 0.001). YNJP-tr effectively reversed these changes, resulting in statistically higher fecal water content than in the model group (*P* < 0.001). Clinically, shorter time to first black stool appearance correlates with improved bowel movement frequency and serves as a marker of constipation relief [26]. The intestinal propulsion rate was a key indicator of intestinal function and constipation severity in mice. The model group exhibited an intestinal propulsion rate of 48.73% ± 6.33%, whereas the YNJP-tr group showed a significant improvement to 72.75% ± 3.26% (Fig. 7E and F), representing a 24.02% increase, which was slightly higher than the rate observed in the DGE-tr group, and far higher than the rate observed in the YNJ-or group (*P* < 0.01). Both the blank patch and the transdermal YNJP (no CVs, containing all 5 herbal components but without CVs) were included as control groups. The blank patch served as a negative control to rule out any effects from the patch matrix or application procedure, whereas the YNJP (without CVs) was used to evaluate the contribution of CVs to the therapeutic efficacy of YNJP. Comparative analysis revealed a statistically significant enhancement (*P* < 0.001) in intestinal propulsion in YNJP-tr mice compared to the model group, demonstrating improved GI motility. The YNJP (without CVs) exhibited weaker effects on fecal water content and intestinal propulsion rate to those of the YNJP patch (no CVs). Overall, these results indicate that YNJP alleviates constipation by increasing fecal water content and promoting GI motility.

**Fig. 8.**
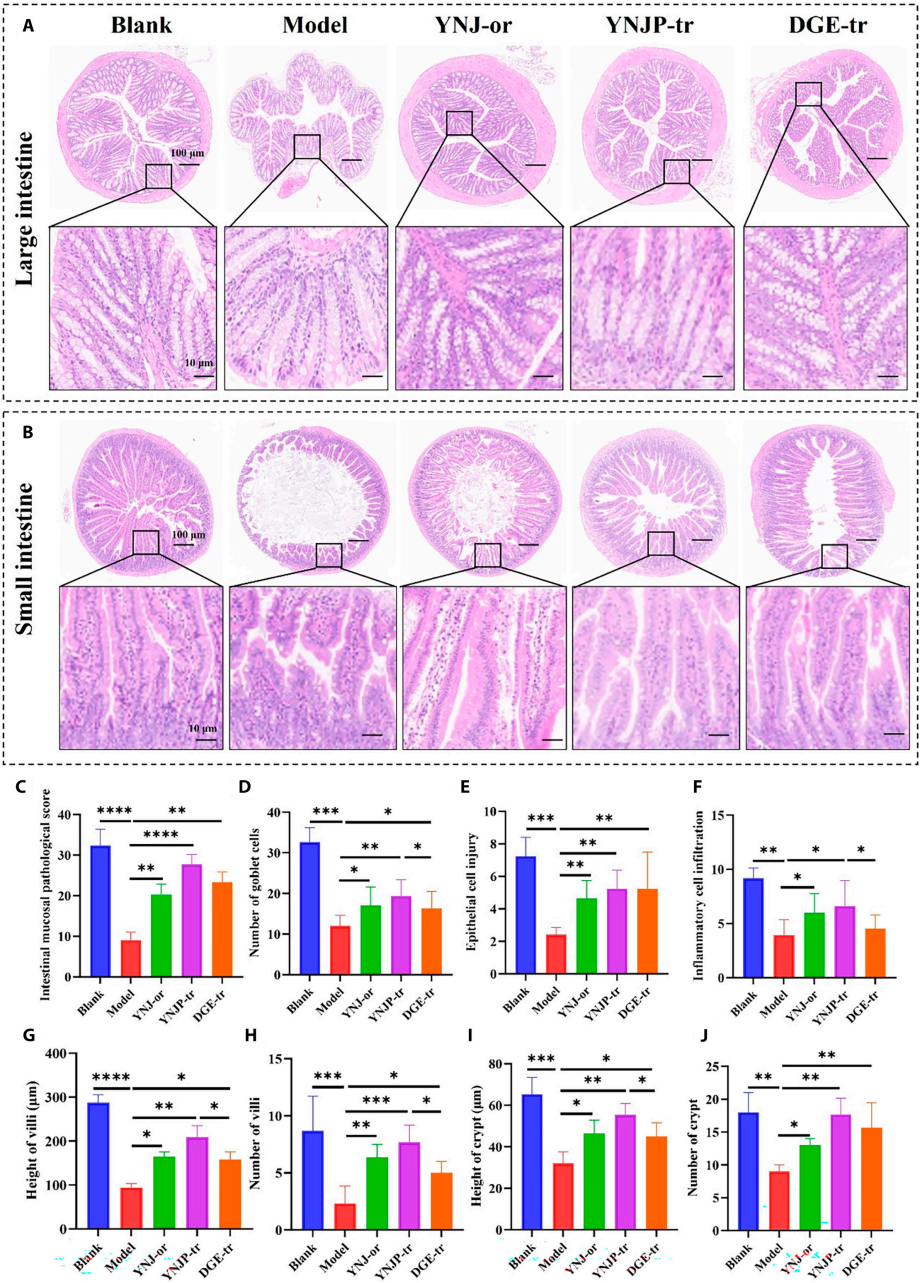
Hematoxylin–eosin (H&E) staining of the alleviation of constipation mediated intestinal barrier damage by YNJP. YNJP in restoring histomorphology of (A) large intestine and (B) small intestine by enhancing barrier function. Scale bar = 100 and 10 μm. (C) Intestinal mucosal pathological score. (D) Number of goblet cells. (E) Epithelial cell damage. (F) Infiltration of inflammatory cells. (G) Height of villi. (H) Number of villi. (I) Height of crypt. (J) Number of crypt.

The pathogenesis of constipation is frequently associated with compromised intestinal barrier integrity, characterized by increased mucosal permeability, inflammatory edema, epithelial cell desquamation, and reduced goblet cell numbers, all of which contribute to disease progression [27]. Histomorphological analysis using H&E staining revealed that intestinal structures in the blank group exhibited normal architecture, with well-organized and closely arranged mucosal villi (Fig. 8A and B). In contrast, constipation induced tissue damage in the GI tract, manifesting as inflammatory cell infiltration, disruption of villous (Fig. 8G and H) and crypt architectures (Fig. 8I and J), and generalized intestinal shrinkage, culminating in impaired colonic contractility. Treatment with YNJP significantly improved histological scores in intestinal tissues (Fig. 8C). The intestinal goblet cells were increased (Fig. 8D). The intestinal structure was largely restored, with no significant epithelial cell degeneration or detachment (Fig. 8E), and minimal inflammatory cell infiltration (Fig. 8F). Moreover, the development of STC is strongly correlated with colonic muscular layer thickness. A reduction in this thickness directly impairs colonic contractility, resulting in 3 main pathological consequences: diminished colonic transit, compromised intestinal motility, and defecation difficulty—all of which contribute to the pathophysiology of functional constipation [28].

### In vivo anti-constipation mechanisms of YNJP by modulating intestinal endocrine and metabolic homeostasis

Constipation induces intestinal damage and inflammation [29], often resulting from the impairment of proteins essential for maintaining intestinal integrity. Water transport system in the intestine, which is closely associated with constipation, is primarily regulated by AQP3, a channel protein that facilitates the excretion of water molecules. Mucin, a high-molecular-weight glycoprotein, contributes to the formation of the intestinal epithelial mucus layer and aids in lubrication by reducing water loss [30]. MUC2, secreted by goblet cells, is essential for the formation of the mucus barrier, supporting normal peristalsis and the lubrication of colonic contents [31,32]. Moreover, zonula occludens-1 (ZO-1), a tight junction protein, is critical for maintaining intercellular adhesion among intestinal epithelial cells and for preserving the integrity of the intestinal epithelial barrier [33].

**Fig. 9.**
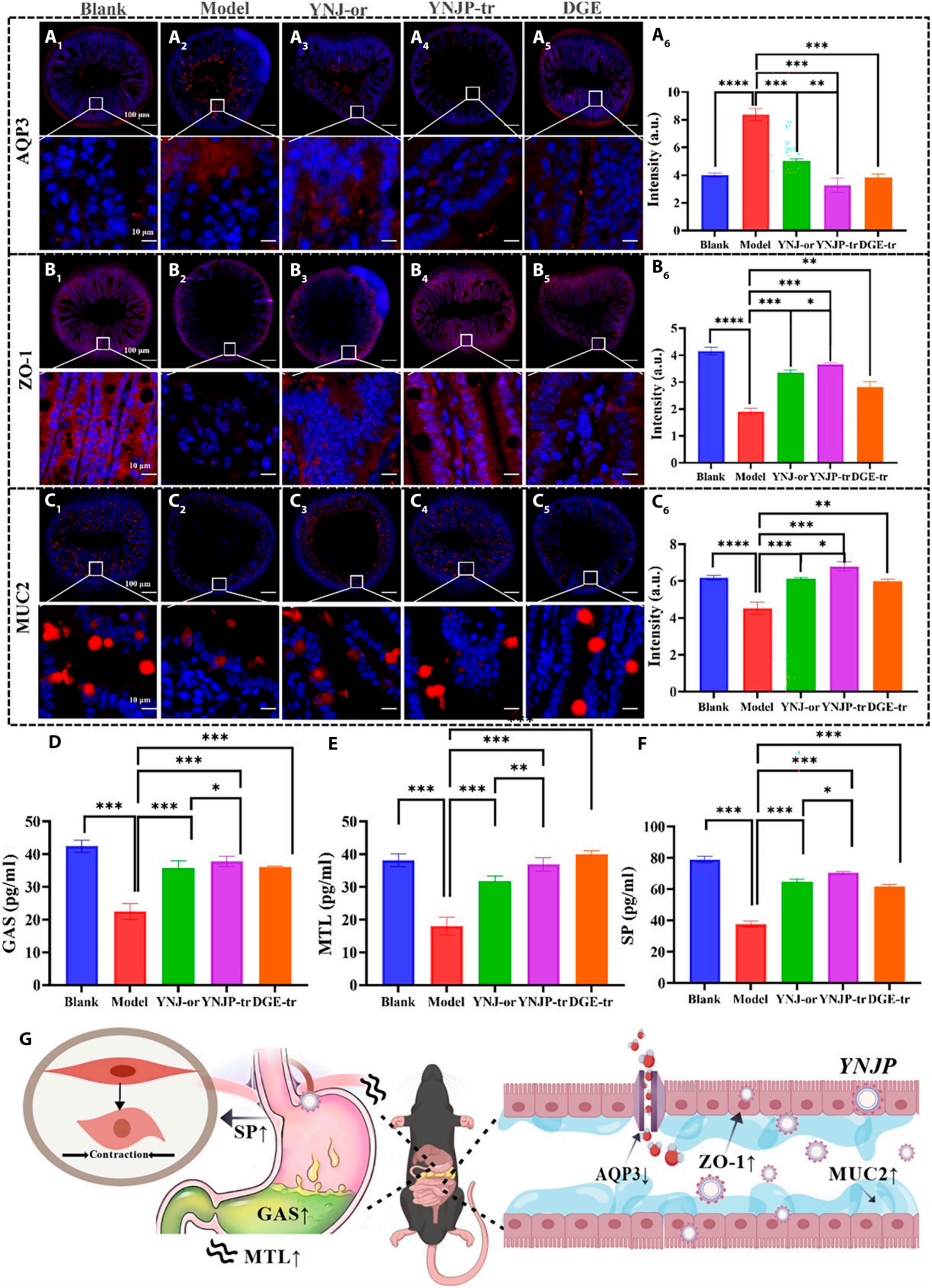
Mechanistic insights into YNJP-mediated modulation of intestinal endocrine and metabolic homeostasis, evaluated via immunofluorescence and ELISA. Immunofluorescence detection of (A_1_ to A_6_) AQP3, (B_1_ to B_6_) MUC2, and (C_1_ to C_6_) ZO-1 in the blank, model, YNJ-or, YNJP-tr, and DGE-tr. The level of endocrine homeostatic transmitters (D) GAS, (E) MTL, and (F) SP. (G) The mechanism diagram of YNJP regulating the secretion of endocrine homeostatic transmitters, up-regulating mucins and aquaporins. *P < 0.05, **P < 0.01, ***P < 0.001, and ****P < 0.0001.

The high degree of glycosylation and exceptionally large molecular weight of MUC2 grant it the ability to form massive polymer networks. Intracellularly, MUC2 undergoes processing, folding, and packaging in the endoplasmic reticulum and Golgi apparatus, resulting in the formation of enormous secretory granules. These large, round or irregular punctate or granular structures nearly fill the entire cytoplasm of the cell. Immunofluorescence staining revealed that AQP3 and MUC2 were distributed throughout all regions of the colonic mucosa, both showing red fluorescence signals (Fig. 9A and C). Furthermore, ZO-1 expression was primarily localized in the upper layer of the colonic mucosa (Fig. 9B), also exhibiting red fluorescence.

Compared with the blank group, the fluorescence intensity of ZO-1, and MUC2 was significantly decreased in the model group (*P* < 0.0001), whereas AQP3 expression was markedly increased (*P* < 0.0001). In contrast, both YNJ-or and YNJP-tr groups showed a marked reduction of expression of AQP3 (Fig. 9A_1_ to A_6_, *P* < 0.001), along with increased expression of ZO-1 (Fig. 9B_1_ to B_6_, *P* < 0.001) and MUC2 (Fig. 9C_1_ to C_6_, *P* < 0.001) in the small intestine relative to the model group, reaching levels comparable to those observed in the DGE treatment group, and the effect is better than oral administration of YNJ (YNJ-or, *P* < 0.05). In summary, these findings indicate that transdermal delivery of YNJP promotes mucus secretion by intestinal goblet cells (via MUC2 up-regulation), inhibits the expulsion of water molecules (via AQP3 down-regulation), and enhances intestinal barrier function (via ZO-1 up-regulation), thereby protecting against foreign substance-induced damage achieving a laxative and anti-constipation effect.

The regulation of GI motility is governed by a complex interplay of neurotransmitters [34]. Receptors for endocrine homeostatic transmitters are abundantly distributed in the colon and play a pivotal role in facilitating intestinal propulsion [35]. Specifically, GAS stimulates gastric secretion and antral contraction, and MTL induces colonic smooth muscle contraction and accelerates content transit, thereby enhancing migrating motor complexes and pepsin secretion [23], while reduced levels of SP are associated with impaired peristalsis and constipation [36]. To investigate the mechanism underlying YNJP-mediated improvements in GI motility, the levels of these neurotransmitters were quantified. As shown in Fig. 9D to F, the model group exhibited significantly lower levels of GAS, SP, and MTL in stomach homogenate compared to the blank group (*P* < 0.001). After treatment, YNJP-tr significantly restored these neurotransmitter levels (*P* < 0.001), reaching values comparable with the DGE-positive control group, and the effect is better than YNJ-or (*P* < 0.05). These findings suggest that EPL disrupts endocrine homeostatic transmitter production, and YNJP promotes GI transport and relieves constipation by modulating intestinal endocrine and metabolic homeostasis (Fig. 9G).

### In vivo anti-constipation mechanisms of YNJP by improving constipation and gut microflora

**Fig. 10.**
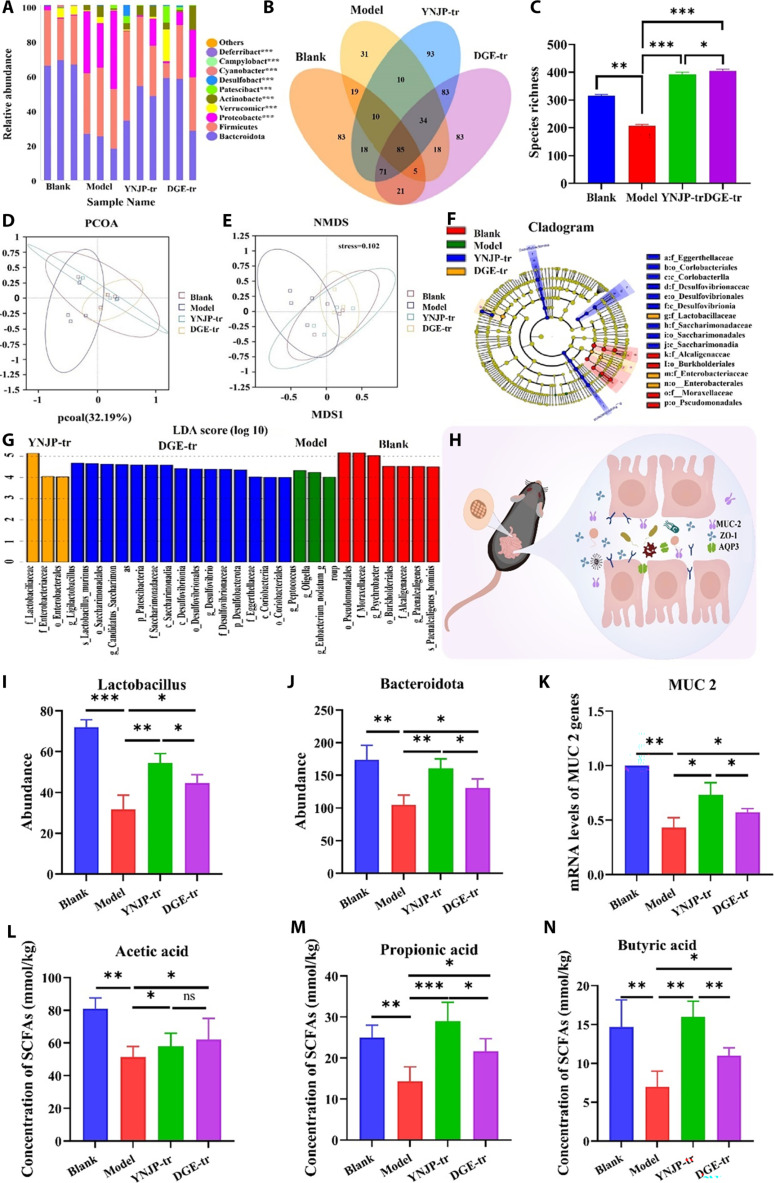
Analysis of gut microbiota composition and short-chain fatty acids (SCFA) levels in mice. Histogram of (A) relative species abundance, (B) Wayne diagram, and (C) charts of the individual groups of species richness. (D) Principal coordinates analysis (PCoA) diagram. (E) Nonmetric multidimensional scaling (NMDS) analysis diagram. (G) Phylogenetic branch plot (phylogenetic distribution) and (F) histogram of LEfSe (LDA effect size) LDA value distribution. (H) Mechanism diagram of gut-associated proteins in the YNJP-treated group. The relative abundance of (I) *Lactobacillus* and (J) *Bacteroidota* in the gut microbiota of mice treated with YNJP. (K) mRNA expression levels of MUC2 gene in the intestines of mice in each group. The release level of (L) acetic acid, (M) propionic acid, and (N) butyric acid in the mice intestine. *P < 0.05, **P < 0.01, and ***P < 0.001.

Modulation of gut microbiota composition has been shown to alleviate the pathological effects of intestinal disorders [37], with substantial evidence confirming distinct microbial profiles between constipated individuals and healthy controls [38]. Notably, the increase of Chao1 and Shannon diversity indices was observed in the treated groups compared to the constipation model group, indicating their therapeutic potential for functional constipation through microbiota regulation [39]. To further investigate the therapeutic effect of YNJP on intestinal peristalsis via microbiota modulation, 16S rRNA sequencing was performed on fecal samples from all experimental groups. The model group showed a significant decrease in overall microbial abundance compared to the blank group, with *Bacteroidota* showing the most significant reduction (Fig. 10J, *P* < 0.01). This phylum exhibits a positive correlation with small intestine transit rate [23]. Similarly, the abundance of *Peptococcus*, a commensal bacterium commonly present in the gut and skin (Fig. 10F), was also decreased. In contrast, both the DGE-tr and YNJP-tr groups showed increased abundance of these taxa (Fig. 10A to C, *P* < 0.001).

In addition, the microbial community structure of the blank group and drug-treated groups exhibited greater similarity to one another (Fig. 10D and E), whereas the model group was clearly distinct. Following transdermal administration of DGE and YNJP, the most prominent shift in gut microbiota composition was an increase in *Lactobacillus* abundance (Fig. 10F, G, and I, *P* < 0.01). As a common intestinal commensal, it exerts protective effects through strong intestinal mucosal adhesion [40], improves flora distribution, antagonizes harmful bacterial colonization, and contributes to the prevention of intestinal disorders (Fig. 10H). Additionally, MUC2 gene expression level in the intestines of the YNJP-tr group was elevated (Fig. 10K, *P* < 0.05), consistent with previous immunofluorescence results (Fig. 9C_1_ to C_6_). Furthermore, we measured the concentrations of acetic acid (Fig. 10L), propionic acid (Fig. 10M), and butyric acid (Fig. 10N) in the mice intestinal contents. The molar concentrations of SCFAs were significantly increased by 1.36- to 1.82-fold in the YNJP-tr group (*P* < 0.05), which are known to be released by *Lactobacillus* and contribute to intestinal protection. Taken together, these findings suggest that YNJP-tr relieves constipation by remodeling the gut microbiota and restoring metabolic homeostasis.

### Safety assessment

To evaluate the safety of YNJP in vitro, its effects on the proliferation of HaCaT and HSF cells were assessed. Results showed that YNJP had negligible effects on cell proliferation (Fig. S3A and B). In addition, histopathological analysis of H&E-stained organs further confirmed the in vivo safety of YNJP, showing no detectable morphological abnormalities or tissue damage (Fig. S3C).

To further evaluate the safety profile of YNJP, a comparative study was conducted against oral YNJ (YNJ-or) at an equivalent dosage. Following transdermal administration of YNJP (YNJP-tr), mercury (Hg) accumulation was significantly reduced across multiple organs, including the liver, kidneys, and brain at the 1- and 30-day time points (Table S1). A comparison of Hg^2+^ accumulation profiles revealed distinctly different kinetics between the 2 administration routes. After oral delivery of YNJ, Hg^2+^ levels increased steadily over time, with the most pronounced accumulation observed in the liver, kidneys, and brain. In contrast, transdermal administration of YNJP resulted in markedly lower Hg^2+^ deposition in these critical organs. Specifically, at the 30-day time point, Hg^2+^ concentrations in the YNJ-or group reached 12.66 μg/g in the kidneys, 7.40 μg/g in the liver, and 0.41 μg/g in the brain, whereas the YNJP-tr group exhibited substantially reduced levels of 3.24, 2.10, and 0.04 μg/g, respectively. These findings demonstrate that the YNJP transdermal system effectively limits systemic mercury exposure and tissue retention in key metabolic and neural organs, underscoring its enhanced safety profile and potential suitability for long-term clinical application.

Additionally, the skin irritation potential of YNJP was evaluated in New Zealand white rabbits. During the 3-day observation period following application, no adverse reactions, such as pigmentation, bleeding, or alterations in skin texture (such as roughness or thinning) were observed at the application site. Throughout both the treatment and recovery phases, no signs of irritation, including erythema or edema, were detected (Fig. S4). The skin irritation score for the YNJP-treated group was calculated as 0.28, indicating only mild irritation (slight redness), with no statistically significant difference compared to the blank control group (*P* > 0.05).

## Discussion

Pediatric constipation is a prevalent GI disorder, affecting approximately 39% of children globally [41,42]. The traditional Chinese medicine preparation YNJ has been conventionally administered orally for constipation treatment. However, to overcome limitations of oral administration for YNJ such as first-pass metabolism [43], we developed an innovative transdermal delivery method [44]. TDDSs represent a noninvasive treatment approach that enhances bioavailability, reduces systemic adverse effects, facilitates self-administration, and improves compliance [45,46]. Thus, TDDS offers distinct advantages over conventional routes such as oral and injectable administration [47].

Furthermore, vesicle-encapsulated cinnabar was incorporated to facilitate transdermal delivery, and a patch-based skeleton design was employed to create a localized drug reservoir on the skin surface. This system regulates drug release, prolonging delivery duration while ensuring sustained and localized release of active compounds [48]. This strategic modification significantly improves administration safety while maintaining, and potentially enhancing therapeutic efficacy, as demonstrated by an 8.13% increase in intestinal propulsion rates (Fig. 7E and F), and its promoting rate is higher than YNJ of oral administration. Moreover, the formulation exerts dual regulatory effects by modulating the expression of key intestinal proteins (including AQP3, ZO-1, and MUC2, Fig. 9A_1_ to C_6_), while concurrently stimulating the secretion of endocrine homeostatic transmitters (such as GAS, MTL, and SP, Fig. 9D to F), and its effect is better than YNJ of oral administration. These findings underscore the clinical utility of YNJP in treating constipation through a “symptom-root co-treatment” therapeutic paradigm.

The present study reveals that transdermal delivery of YNJ significantly reduces systemic mercury accumulation compared with oral administration, underscoring its advantage in mitigating the toxicity risks inherent to cinnabar-containing formulations. However, it is important to interpret this finding within the broader therapeutic landscape of constipation management. First-line oral agents for pediatric constipation, such as polyethylene glycol, are characterized by minimal intestinal absorption and exceptionally low systemic toxicity. In such cases, the safety advantage of transdermal delivery over oral administration is less pronounced, as the oral route itself does not pose significant systemic risks. Therefore, the safety benefits of the YNJP transdermal system should be understood primarily in comparison with oral YNJ, the traditional formulation, rather than as a general advantage over all oral constipation therapies. The key value of transdermal delivery in this context lies in its ability to enable the safe application of a traditionally oral Chinese medicinal formula that contains potentially toxic minerals, while also offering additional benefits such as improved pediatric compliance, avoidance of GI irritation, and the potential for localized intestinal targeting via umbilical application.

The therapeutic effects of YNJP on constipation can be attributed to its synergistic regulation of 3 key intestinal barrier components: AQP3, MUC2, and ZO-1. AQP3 is a water channel protein primarily responsible for water reabsorption in the colon. In constipation, AQP3 expression is often up-regulated, leading to excessive water extraction from feces and consequent dry, hard stools. YNJP significantly down-regulated AQP3 expression (Fig. 9A_1_ to A_6_), thereby reducing colonic water reabsorption and increasing fecal water content. MUC2, the major gel-forming mucin secreted by goblet cells, constitutes the intestinal mucus layer that lubricates the intestinal lumen and protects the underlying epithelium from mechanical and microbial insults. Constipation is associated with reduced MUC2 expression and mucus layer thinning. YNJP markedly up-regulated MUC2 expression (Fig. 9C_1_ to C_6_), restoring mucus secretion and facilitating smoother intestinal transit. ZO-1, a key tight junction protein, maintains the integrity of the intestinal epithelial barrier. Impaired ZO-1 expression increases paracellular permeability, contributing to inflammation and barrier dysfunction in constipation. YNJP treatment enhanced ZO-1 expression (Fig. 9B_1_ to B_6_), reinforcing epithelial integrity and reducing barrier disruption. Collectively, YNJP exerts a multitarget modulation on the intestinal barrier by simultaneously decreasing AQP3-mediated water reabsorption, increasing MUC2-mediated mucus lubrication, and enhancing ZO-1-mediated epithelial integrity. This coordinated regulation addresses 3 distinct yet interrelated facets of constipation pathophysiology, namely, stool hydration, intestinal lubrication, and barrier protection, culminating in a comprehensive therapeutic outcome that extends beyond mere prokinetic activity.

We systematically evaluated the efficacy of YNJP in enhancing GI motility using a juvenile murine model of drug-induced constipation. YNJP alleviates constipation through gut microbiota modulation, notably enriching *Bacteroidota* and *Lactobacillus* species (Fig. 9I and J), which enhance intestinal protection. Additionally, YNJP significantly increased the production of SCFAs, including acetic acid, propionic acid, and butyric acid by 1.36- to 1.82-fold (Fig. 9L to N). It also stimulates the secretion of 5-hydroxytryptamine from enterochromaffin cells, thereby activating colonic smooth muscle contraction [49] and improving intestinal motility. Additional mechanisms include restoring microbial homeostasis, attenuating intestinal inflammation, and enhancing mucosal barrier function [50].

Notably, the concurrent modulation of gut microbiota and endocrine homeostatic transmitters by YNJP raises the possibility of a functional interaction between these 2 regulatory systems. SCFAs, including acetic, propionic, and butyric acids, are key metabolites produced by *Lactobacillus* and other commensal bacteria. Accumulating evidence indicates that SCFAs can stimulate enteroendocrine cells via activation of G protein-coupled receptors such as GPR41 and GPR43, thereby promoting the secretion of GI hormones including GAS, MTL, and SP. In the present study, YNJP treatment significantly increased the abundance of *Lactobacillus* and elevated SCFA levels (Fig. 10I and L to N), accompanied by enhanced secretion of GAS, MTL, and SP (Fig. 9D to F). It is plausible that the YNJP-induced enrichment of SCFA-producing bacteria contributes, at least in part, to the stimulation of enteroendocrine cells, thereby promoting GI motility and alleviating constipation. This potential microbiota–endocrine crosstalk suggests that YNJP may exert its therapeutic effects not only through direct regulation of intestinal barrier proteins but also through indirect modulation of the gut microbiota–host endocrine axis. Such a dual mechanism aligns with the multitarget characteristics of Chinese medicinal formulas and provides a more comprehensive understanding of how YNJP restores intestinal homeostasis in constipation.

It should be noted that the present study employed adult mice (6 to 8 weeks old) to establish the transdermal delivery platform and evaluate the core mechanisms of YNJP. While this approach allowed for a rigorous proof-of-concept assessment of vesicle-mediated transdermal delivery and its therapeutic effects on STC, the use of adult animals does not fully recapitulate the immature GI and integumentary physiology of the pediatric population (0 to 6 years) for whom YNJ is traditionally indicated. However, transdermal administration inherently bypasses GI absorption and first-pass metabolism, thereby mitigating some of the age-related differences in drug disposition. Future studies utilizing juvenile or weanling animal models are warranted to further validate the translational potential of YNJP for pediatric constipation.

In summary, the TDDS demonstrates a particular promising therapeutic strategy for pediatric applications by maintaining efficacy while improving biosafety. Nevertheless, several technical challenges require further investigation, particularly regarding drug release efficiency and the penetration dynamics of certain herbal constituents in transdermal formulations [51,52].

## Conclusion

The transdermal delivery of Chinese medicinal formula offers a safer and more effective novel strategy for the localized treatment of intestinal disorders, holding particular promise for pediatric applications. The efficacy of YNJP in alleviating pediatric constipation through multitarget regulation of the intestinal microenvironment was shown, including intestinal hydration enhancement, mucus barrier function improvement, and intestinal endocrine homeostasis modulation. Furthermore, the remodeling of the gut microbiota and metabolic homeostasis by YNJP was identified for the first time as the new mechanism of YNJ in treating intestinal disorders.

## Ethical Approval

All animal experimental procedures were performed in obedience to the guidelines and protocols of the Animal Experimental Ethics Committee of Zhejiang University (ZJU20170733).

## Data Availability

The data that support the findings of this study are available from the corresponding authors upon reasonable request.
