# Supplementary material for: Transdermal Delivery of Chinese Medicinal Formula Mitigates Pediatric Constipation by Modulating Intestinal Endocrine and Metabolic Homeostasis
Source: Biomater Res. 2026 Jun 10;30:0374. doi: 10.34133/bmr.0374 (PMC13250285; doi:10.34133/bmr.0374)
Supplement: Supplementary 1 — Figs. S1 to S4 Tables S1 to S6 [file bmr.0374.f1.docx]

**Transdermal delivery of Chinese medicinal formula mitigates** **pediatric constipation** **by** **modulating intestinal endocrine and metabolic homeostasis**

Fengyuan Song^1^^,2#^, Yunhao Ren^2#^, Ming Zhu^2^, Yuling Liu^4^, Siping Wei^1*^, Hui Li^4,5*^, Lihua Peng^2^^,3*^

^1^ School of Pharmacy, Southwest Medical University, Luzhou 646009, Sichuan, PR China.

^2^ College of Pharmaceutical Sciences, Zhejiang University, Hangzhou 310058, Zhejiang, PR China.

^3^ State Key Laboratory of Quality Research in Chinese Medicine, Macau University of Science and Technology, Macau, P. R. China

^4^ Institute of Chinese Materia Medica, China Academy of Chinese Medical Sciences, Beijing 100700, PR China.

^5^ Institute of Traditional Chinese Medicine Health Industry, China Academy of Chinese Medical Sciences, Nanchang 330115, Jiangxi, PR China.

^#^ Fengyuan Song and Yunhao Ren contributed equally to this work.

**Corresponding authors at:**

**Lihua Peng, Ph.D, Professor**

E-mail address: [lhpeng@zju.edu.cn](mailto:lhpeng@zju.edu.cn)

Tel/Fax: 86-571-88981231

College of Pharmaceutical Sciences, Zhejiang University, 866# Yuhangtang Road, Hangzhou 310058, P.R. China.

**Siping Wei, Ph.D, Professor** E-mail address: swei1225@swmu.edu.cn

School of Pharmacy, Southwest Medical University, Luzhou 646009, Sichuan, PR China.

**Hui Li, Ph.D, Professor** E-mail address: lihuiyiren@163.com

Institute of Chinese Materia Medica, China Academy of Chinese Medical Sciences, Beijing 100700, PR China.

**
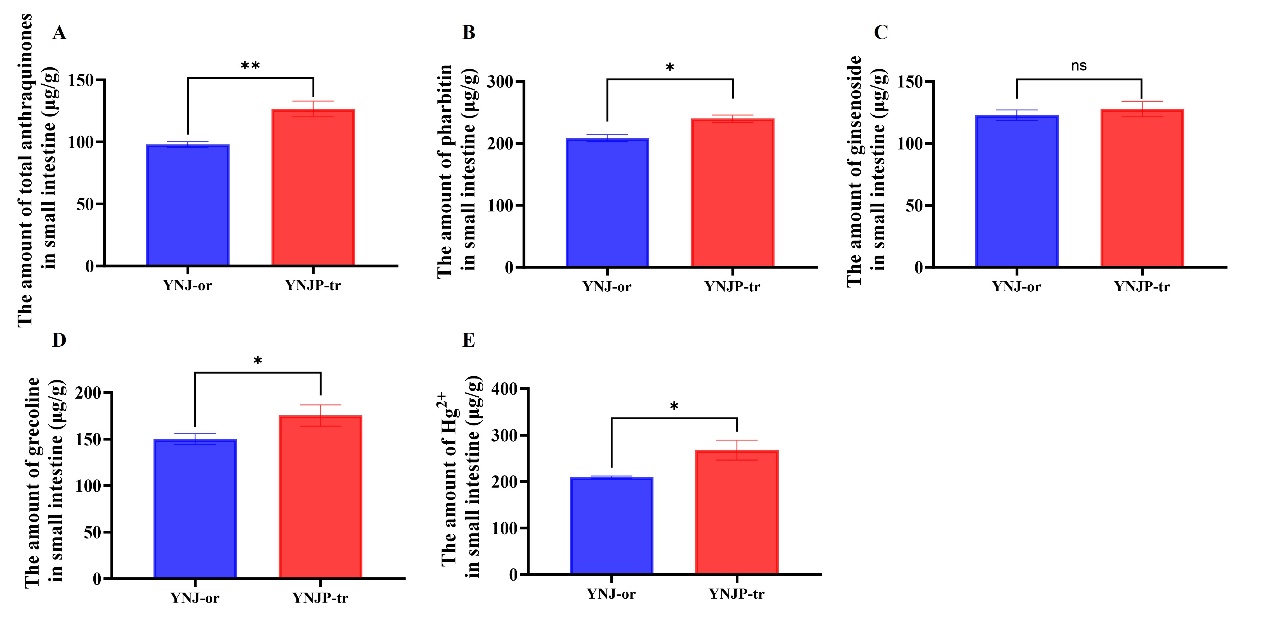
**

**Figure S1.** In the YNJ-or and YNJP-tr groups, the contents of (A) total anthraquinones, (B) pharbitin, (C) ginsenoside, (D) arecoline, and (E) Hg^2+^ in the small intestine.


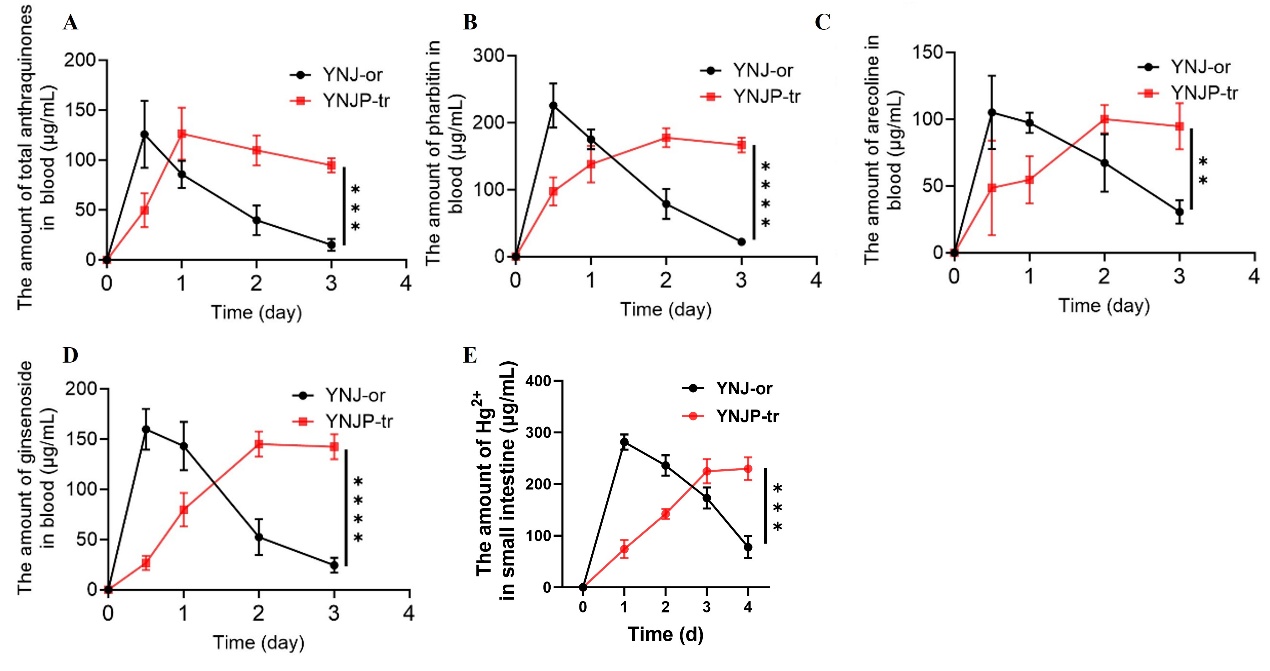


**Figure S2.** Plasma concentrations *in vivo* of (A) total anthraquinones, (B) pharbitin, (C) arecoline, (D) ginsenoside, and (E) Hg^2+^ within 0-3 d. ** *p* < 0.01, *** *p* < 0.001, and **** *p* < 0.0001.


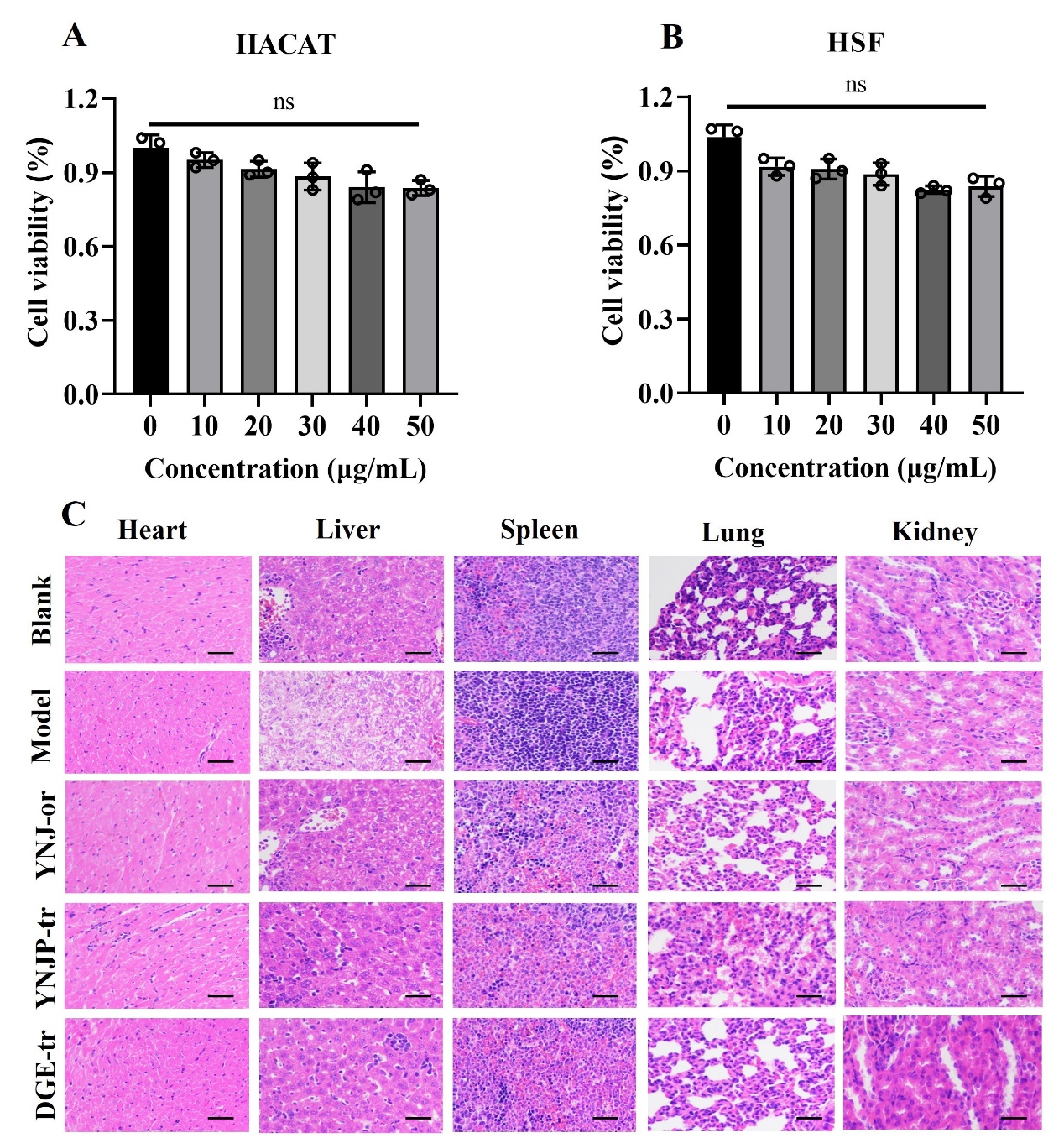


**Figure S3.** **Safety assessment of YNJP**. Cell viability of the YNJP on (A) HACAT and (B) HSF skin cells. Hematoxylin-eosin staining (H&E) staining of heart, liver, spleen, lung, and kidney in blank group, model group, YNJ-or, YNJP-tr, DGE-tr, and scale bar = 50 μm. ^ns^ *p* > 0.05.


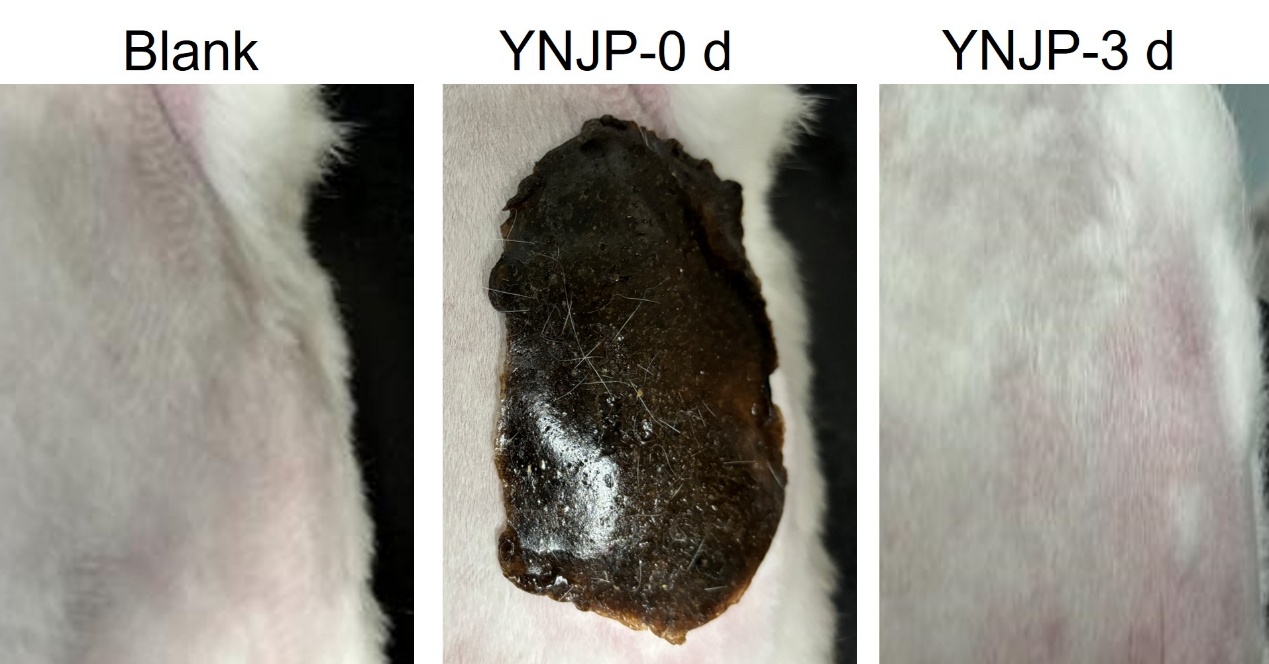


**Figure S4.** The skin irritation intensity of New Zealand white rabbit skin treated with YiNianJin patch (YNJP) for 3 d.

**Table S1**. Comparison of short- and long-term toxicities: oral delivery of YiNianJin (YNJ-or) and transdermal delivery of YiNianJin patch (YNJP-tr).

| Organs | Delivery methods | 1 d (Hg^2+^, μg/g) | 30 d (Hg^2+^, μg/g) | Folds |
| --- | --- | --- | --- | --- |
| Liver | YNJ-or | 0.45 ± 0.21 | 7.40 ± 1.32 | 16.4 |
|  | YNJP-tr | 0.29 ± 0.04 | 2.10 ± 0.35 | 7.2 |
| Kidneys | YNJ-or | 0.72 ± 0.30 | 12.66 ± 2.40 | 17.6 |
|  | YNJP-tr | 0.33 ± 0.32 | 3.24 ± 1.10 | 9.8 |
| Brain | YNJ-or | 0.02 ± 0.01 | 0.41 ± 0.08 | 20.5 |
|  | YNJP-tr | 0.01 ± 0.01 | 0.04 ± 0.01 | 4.0 |

**Table S2.** Gradient elution procedure of HPLC detection

| Time(min) | Mobile phase A (%) | Mobile phase B (%) |
| --- | --- | --- |
| 0-35 | 19 | 81 |
| 35-55 | 19→29 | 81→71 |
| 55-70 | 29 | 71 |
| 70-100 | 29→40 | 71→60 |

**Table S3.** Microwave digestion program

| Step | Heating up time /min | Control temperature /℃ | Hold time /min |
| --- | --- | --- | --- |
| 1 | 10 | 120 | 10 |
| 2 | 10 | 180 | 60 |

**Table S4.** The working parameters of ICP

| Parameters | Value | Parameters | Value |
| --- | --- | --- | --- |
| RF power/(W) | 1550 | Peristaltic pump speed/(r/min) | 6.0 |
| Plasma flow rate/(L/min) | 15 | Scanning method | Jumping Peak |
| Atomizing gas flow rate/(L/min) | 0.88 | Repeated collection times | 3 times |
| Soft air (flow rate/(L/min) | 1.10 | Solution stability time/s | 40 |
| Atomization chamber temperature/℃ | 2 | Integral time/s | 0.3 (Cd 1.0) |
| Collision masking gas flow rate/(L/min) | 4.0 | Oxide/% | 0.69 |
| Sampling depth/mm | 8.0 | Double charge/% | 1.02 |

**Table S5**. The primers of MUC 2 genes

| Genes | Forward primers (5’-3’) |  | | Reverse primers (5’-3’) |
| --- | --- | --- | --- | --- |
| MUC 2 | GCCGAGGCTCCTACAAGG | |  | GGCATCGCTCTTCTCAATG |
| β-actin | AGAGGGAAATCGTGCGTGAC | |  | CAATAGTGATGACCTGGCCGT |

**Table 6**. Mass spectrometric parameters for analysis of SCFAs

| Number | Target compounds | Retention time/min | Quantitative ion (*m/z*) | Qualitative ion (*m/z*) |
| --- | --- | --- | --- | --- |
| 1 | acetic acid | 6.86 | 43 | 60, 45 |
| 2 | propionic acid | 7.94 | 43 | 73, 45 |
| 3 | butyric acid | 9.00 | 60 | 73, 41 |
